# Contribution of Mitochondria to Insulin Secretion by Various Secretagogues

**DOI:** 10.1089/ars.2021.0113

**Published:** 2022-05-06

**Authors:** Petr Ježek, Blanka Holendová, Martin Jabůrek, Andrea Dlasková, Lydie Plecitá-Hlavatá

**Affiliations:** Department of Mitochondrial Physiology, Institute of Physiology of the Czech Academy of Sciences, Prague, Czech Republic.

**Keywords:** pancreatic β-cell metabolism, insulin secretion, redox signaling, mitochondrial Ca^2+^ transport, branched-chain ketoacid oxidation, fatty acid-stimulated insulin secretion, ATP-sensitive K^+^ channel, TRPM channels, GLP-1

## Abstract

**Significance:**

Mitochondria determine glucose-stimulated insulin secretion (GSIS) in pancreatic β-cells by elevating ATP synthesis. As the metabolic and redox hub, mitochondria provide numerous links to the plasma membrane channels, insulin granule vesicles (IGVs), cell redox, NADH, NADPH, and Ca^2+^ homeostasis, all affecting insulin secretion.

**Recent Advances:**

Mitochondrial redox signaling was implicated in several modes of insulin secretion (branched-chain ketoacid [BCKA]-, fatty acid [FA]-stimulated). Mitochondrial Ca^2+^ influx was found to enhance GSIS, reflecting cytosolic Ca^2+^ oscillations induced by action potential spikes (intermittent opening of voltage-dependent Ca^2+^ and K^+^ channels) or the superimposed Ca^2+^ release from the endoplasmic reticulum (ER). The ATPase inhibitory factor 1 (IF1) was reported to tune the glucose sensitivity range for GSIS. Mitochondrial protein kinase A was implicated in preventing the IF1-mediated inhibition of the ATP synthase.

**Critical Issues:**

It is unknown how the redox signal spreads up to the plasma membrane and what its targets are, what the differences in metabolic, redox, NADH/NADPH, and Ca^2+^ signaling, and homeostasis are between the first and second GSIS phase, and whether mitochondria can replace ER in the amplification of IGV exocytosis.

**Future Directions:**

Metabolomics studies performed to distinguish between the mitochondrial matrix and cytosolic metabolites will elucidate further details. Identifying the targets of cell signaling into mitochondria and of mitochondrial retrograde metabolic and redox signals to the cell will uncover further molecular mechanisms for insulin secretion stimulated by glucose, BCKAs, and FAs, and the amplification of secretion by glucagon-like peptide (GLP-1) and metabotropic receptors. They will identify the distinction between the hub β-cells and their followers in intact and diabetic states. *Antioxid. Redox Signal*. 36, 920–952.

## Introduction

### Mitochondria as metabolic and redox hub

Mitochondria have been recognized for seven decades as the metabolic and redox hub, not only providing cells with ATP but also with a plethora of metabolites and signaling mechanisms. Mitochondria cannot be ignored in the majority of studies working toward understanding physiological and pathological mechanisms at the subcellular level. For pancreatic β-cells, the ultimate physiological role of mitochondria lies in the notoriously known elevation of ATP synthesis upon glucose-stimulated insulin secretion (GSIS). However, mitochondrial redox signaling is one of its recently discovered roles (104, 194), as well as the transport of Ca^2+^ across the inner mitochondrial membrane (IMM), synchronized with Ca^2+^ oscillations evoked by action potential firing, which are caused by the predominantly intermittent opening of voltage-dependent Ca^2+^ channels (Ca_V_); in rodents, these are mostly L-type channels (Ca_L_) (31, 91, 210, 212, 213).

The additional Ca^2+^ release is superimposed onto the primary Ca^2+^ oscillations incoming from the endoplasmic reticulum (ER) (49, 280) and other Ca^2+^ stores, such as insulin granule vesicles (IGVs) or lysosomes. By stimulating matrix dehydrogenases and adenylyl cyclase, the elevated matrix Ca^2+^ plays an important amplifying role during the first and second GSIS phase and during amplification mechanisms of insulin secretion, notably in incretin- (glucagon-like peptide [GLP-1]- and gastric inhibitory peptide [GIP]-) and metabotropic receptor signaling (71, 218).

We found that insulin secretion stimulated by branched-chain ketoacids (BCKAs) (194) and partly by fatty acids (FAs) (98, 103) essentially relies on mitochondrial retrograde redox signaling. Due to the relatively low content of cytosolic glutathione (17, 135–137, 270), the redox milieu of pancreatic β-cells promotes the signal spreading from mitochondria up to the targets within the plasma membrane, which can further switch-on Ca_V_ opening and action potential firing, followed by IGV exocytosis. It is unknown whether the redox signal spreading is enabled by a H_2_O_2_ diffusion or by a redox relay, for example, *via* peroxiredoxins, thioredoxins, or glutaredoxins, abundant in pancreatic β-cells (97, 204). In any case, β-cells appear to be a perfect redox system, ideally suited for redox signal conduction (105, 274). Nevertheless, the redox state is highly compartmentalized (17, 208).

Also, the specific metabolism of β-cells under fasting and fed conditions contributes *via* changes and the concomitant effects of NADH/NADPH homeostasis, as well as *via* the transport of various specific metabolites, for example, coenzyme A-esters (CoA-esters) of FAs, malonyl-CoA, and long-chain acyl-CoA as stimulating insulin secretion (196), formed from the matrix acetoacetate exported to the cytosol (52).

### ATP-sensitive K^+^ channel as prerequisite for triggering of GSIS

**FIG. 1.**
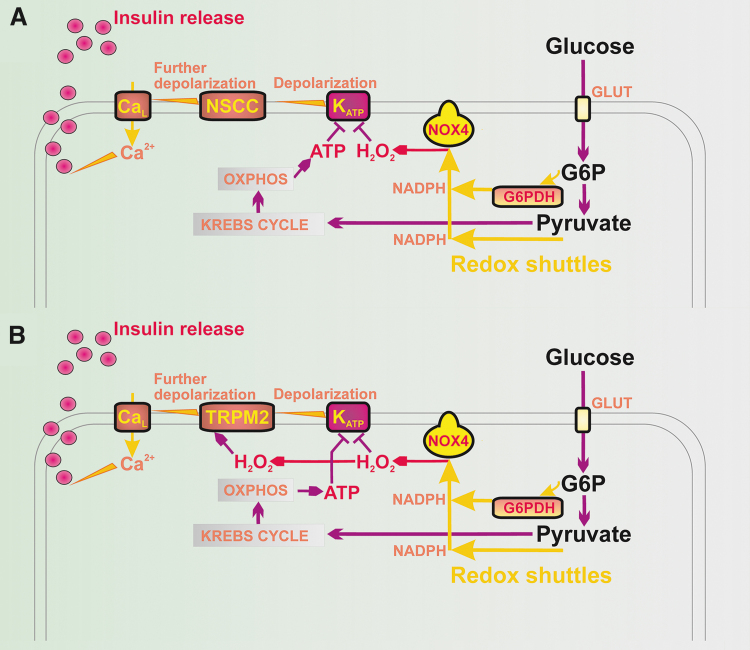
**Redox signaling triggers GSIS in parallel with ATP. (A)** Hypothetical model based on Plecita-Hlavata *et al*. (194), in which only K_ATP_ is redox-regulated; K_ATP_ closure is only possible when H_2_O_2_ and ATP are elevated. **(B)** Extended hypothetical model, in which TRPM2 is also redox-activated. For an explanation, see the Introduction section and the Plasma Membrane Depolarization in Pancreatic β-Cells section. G6PDH, glucose-6-phosphate dehydrogenase; GSIS, glucose-stimulated insulin secretion; K_ATP_, ATP-sensitive K^+^ channel; TRPM, transient receptor potential melastin.

Recently, we reported that GSIS essentially relies on the physiological cytosolic redox signaling provided by H_2_O_2_ produced by NADPH oxidase 4 (NOX4) upon glucose intake, followed by the branching of the glucose-6-phosphate (G6P) flux toward the pentose phosphate pathway (PPP) (Fig. 1) (194). The two PPP enzymes produce NADPH, and the elevation of their activities causes an instant elevation of H_2_O_2_ formation by NOX4. This new paradigm of the requirement of increased ATP plus increased H_2_O_2_ for insulin secretion in response to glucose was concluded from experiments in which NOX4-knockout mice (NOX4KO) or mice with NOX4, ablated specifically in pancreatic β-cells (NOX4βKO mice), exhibited a completely suppressed first GSIS phase, whereas the second phase was only moderately attenuated (194).

The first phase was rescued by NOX4 overexpression in pancreatic islets (PIs) isolated from NOX4βKO mice or by H_2_O_2_ addition (194). Moreover, the ATP-sensitive K^+^ channel (K_ATP_) (8) could not be closed after the glucose addition to the patch-clamped INS-1E cells silenced for NOX4 (194). In contrast, INS-1E cells having vestigial ATP synthase lacking DAPIT and thus having crippled ATP synthesis still maintained GSIS (134).

The textbook paradigm stressed the key role of glucose triggering of the first GSIS phase [reviewed, *e.g*., in Refs. (104, 210)]. Glycolysis followed by the oxidative phosphorylation (OXPHOS) and elevated synthesis of ATP has been considered to be the only required condition, similar to the exclusive role of K_ATP_. In The Synergy of Membrane Channels section, we will discuss that even 100% closure of the ensemble of K_ATP_ is not enough for GSIS triggering. In contrast, certain forms of the maturity-onset diabetes of the young (MODY), that is, of monogenic type of diabetes mellitus, are exemplar cases supporting the important role of K_ATP_.

Thus, homogeneous mutations in *Kcnj11* (a gene encoding the KIR6.2 subunit of K_ATP_, when the KIR6.2 tetramer forms the physical channel) and more heterogeneous mutations in the *Abcc8* gene (encoding the regulatory subunits sulfonylurea receptor 1 [SUR1]) reduce the ability of ATP to cause channel closure (9). These mutations impair ATP binding at KIR6.2 or how ATP binding translates into the pore closure, respectively (128, 161, 232). They may enhance MgADP activation of SUR1 by increasing the affinity of the nucleotide-binding domains for nucleotides (185). Both mutations can increase the unliganded channel open probability, which leads to a decrease in both ATP and possible sulfonylurea block (11, 199). However, note also that in different MODY types different gene mutations occur (*e.g*., glucokinase gene *GCK* or genes encoding transcription factors *HNF1α/4α*, *PDX*), all affecting insulin secretion.

In this review, all the above-described aspects of the mitochondrial physiology of pancreatic β-cells will be discussed, including the “logical summation” principle of metabolic plus redox stimulation for the mitochondrial source of H_2_O_2_, which besides GSIS plays an essential role in insulin secretion stimulated by BCKAs (194) and partially by FAs (103). Without detailed knowledge of the redox system of pancreatic β-cells and their sensing of glucose or other secretagogues, the health issues that develop due to type 2 diabetes (107, 248) cannot be understood. Hence, we collected up-to-date knowledge on mitochondria as key players in the physiology of pancreatic β-cells and the pathology of diabetes.

## Mechanisms of Insulin Secretion

### Plasma membrane depolarization in pancreatic β-cells

#### The synergy of membrane channels

Quite recently, an explanation was suggested as to why a 100% closed K_ATP_ population is still insufficient to induce the threshold depolarization (−50 mV) of plasma membrane potential (*V*p), required for Ca_V_ opening and thus for switching on action potential firing (119, 221). *V*p should be shifted far more than enabled by the 100% K_ATP_ closure alone. This additional *V*p shift can be facilitated by numerous “synergic” channels (210), namely by the opening of nonspecific calcium channels (NSCCs), such as transient receptor potential melastin (TRPM) channel-2 (TRPM2) (79, 123, 210, 285), or by the concerted action of chloride channels (45). Moreover, TRPM2 channels are activated by H_2_O_2_ (79, 84, 123, 223), hence they could theoretically also contribute to the “logical sum” of the redox plus metabolic (ATP) signal.

**FIG. 2.**
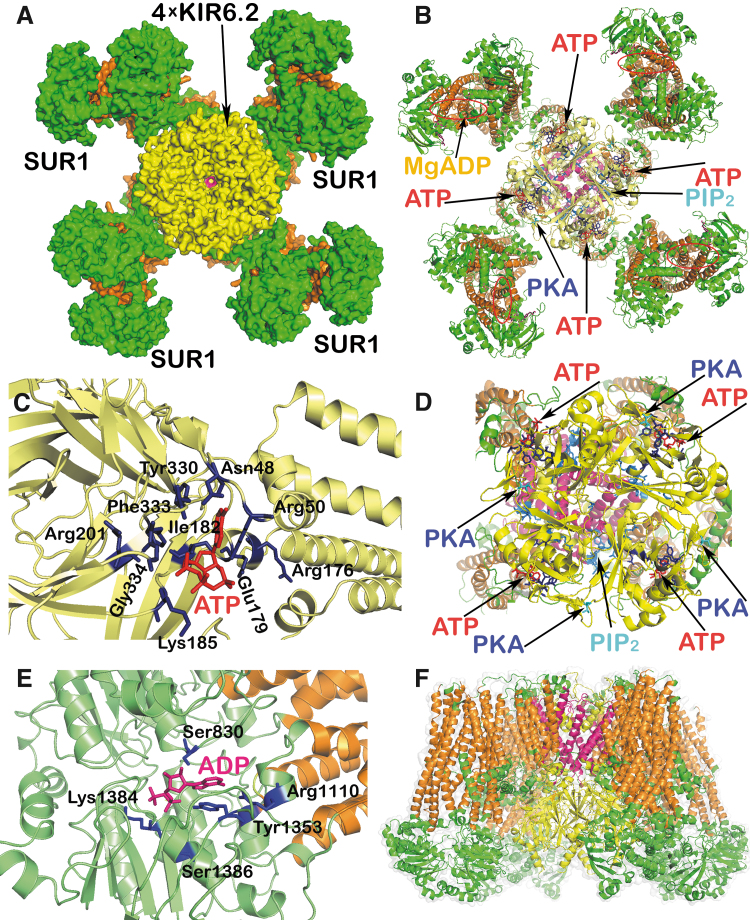
**K_ATP_ channel structure and regulation.** Structures of both types of subunits of hetero-octameric K_ATP_ have been resolved, that is, the SUR1 (a product of *Abcc8* gene) and the pore-forming subunit, a potassium inward rectifier, KIR6.2 (*Kcnj11* gene) (101, 142, 159, 210). The displayed model of K_ATP_ channel was derived from the cryo-EM structure of the pancreatic ATP-sensitive K^+^ channel SUR1/Kir6.2 in the presence of ATP and glibenclamide, pdb code 5twv (159), and cryo-EM structure of human K_ATP_ bound to ATP and ADP in quatrefoil form, pdb code 6c3o (131). The structure was visualized using the PyMOL Molecular Graphics System, Version 1.8 Schrödinger, LLC. **(A)** K_ATP_ channel from the intracellular site. **(B)** Visualization of the ATP and PIP_2_ binding sites on the Kir6.2 subunits, Mg^2+^-ADP binding pocket on the SUR1 subunits, and PKA interaction site within the Kir6.2. **(C)** Detail of the binding domain for ATP (in *red*) on the Kir6.2 subunit with interacting amino acid residues (in *dark blue*). **(D)** Detail of the Kir6.2 with ATP and PIP_2_ binding domains and PKA interaction site. **(E)** Detail of the SUR1 Mg^2+^-ADP binding site (in *pink*) with interacting amino acid residues (in *dark blue*). **(F)** Side view of the K_ATP_ channel; color coding: intracellular regions of Kir6.2 subunits in *yellow*, transmembrane domains in *dark pink*; intracellular domains of SUR1 subunits in *green*, transmembrane helices in *orange*. Four Kir6.2-subunits cluster together, forming the core of the ∼18 × 13 nm entire structure (166). The cytoplasmic Kir6.2 surface contains the ATP-binding site, implicated in the channel closing, exposed 2 nm below the membrane. An overlapping PIP_2_ binding site stabilizes the open state. Upon PIP_2_ release, the open probability decreases (14, 166, 234). The channel is closed as soon as the first ATP-binding site is occupied, one of four ATP-binding sites (179). Sensitivity to PIP_2_ is regulated by the palmitoylation of Cys166 (277). Mg^2+^-free ATP decreases the duration of channel openings, while periods of closing are longer (36), whereas MgADP acts in the other direction (118). Artificial K_ATP_ openers (diazoxide) and K_ATP_ blockers, such as sulfonylureas (glibenclamide bound to SUR1), act independent of high ATP (233). Of the eight sites of four SUR1 subunits, each one bears an MgATP- plus MgADP-binding site. At the NBF1 of the former, MgATP is hydrolyzed to MgADP, activating K_ATP_ at NBF2 and increasing the ATP-sensitive K^+^-conductance. This provides lower excitability and sensitivity to ATP inhibition (179). Already 5–15 μ*M* ATP (IC_50_ 10 μ*M*; ∼25 μ*M* with Mg^2+^) closes the channel in inside–out patches, in which the medium affects the cytosolic side (35, 262). In contrast, in intact resting β-cells, much higher [ATP] is required to close K_ATP_. IC_50_ of ∼0.6 m*M* was found for the whole-cell patch-clamp mode (242). This low sensitivity is adjusted by the PKA phosphorylation of Thr224 (144) and Ser372, which increases the K_ATP_ open probability (16). Any further closing only occurs at higher [ATP] or even hypothetically requires H_2_O_2_. NBF, nucleotide-binding fold; PIP_2_, phosphatidylinositol 4,5-bisphosphate; PKA, protein kinase A; SUR1, sulfonylurea receptor 1.

These “synergic” channels provide a small background inward current that cannot depolarize with an open K_ATP_, but it is able to do so with a predominantly closed K_ATP_ ensemble since the NSCC conductance is then comparable to the small conductance provided by the remaining open K_ATP_ channels (K_ATP_ properties, Figs. 1 and 2). Also, the indirect inhibition of K_ATP_ by H_2_O_2_ was observed in smooth muscle cells (283).

The plasma membrane of β-cells possesses up to 60 channels belonging to 16 ion channel families (210, 280), with a distinct pattern in humans (101). Since the ∼130 m*M* [K^+^]_in_ concentration inside the β-cell is much greater than outside ([K^+^]_out_ ∼5 m*M*), there would be an equilibrium resting *V*p^equi^ of −82 mV, if only there was a K^+^-channel conductance. The actual *V*p^Resting^ is −75 mV (49); hence, NSCCs and other channels should provide this shift since NSCCs conduct any Na^+^, Ca^2+^, and K^+^. Evidence came from the observed depolarization reversal after the withdrawal of Ca^2+^ and Na^+^ at a 10 m*M* concentration of glucose ([glucose]) in mouse β-cells (213). Without this NSCC conductance, the established *V*p would only be equal to *V*p^equi^ and the shift to −50 mV, required for Ca_V_ opening (212), would not take place, despite the 100% closed K_ATP_ ensemble (19, 119, 221, 239, 249, 280).

Besides there being a redox-activated TRPM2 channel (79, 123, 223), there are also the Ca^2+^- and cAMP-activated TRPM4 and TRPM5 channels in rodent β-cells (119), plus the heat-activated transient receptor potential vanilloid 1 (TRPV1, capsaicin receptor), TRPV2, TRPV4, or transient receptor potential canonical 1 and 3 (TRPC1, TRPC3) channels. TRPC3 provides an additional shift upon G-protein-coupled receptor (GPR) 40 receptor activation by FAs (276). Similarly, Cl^−^ channels (SLC12A, SLC4A, SlC26A, GABA_A_, GABA_B_, and glycine receptor Cl^−^-channel) (45) and others (210) were implicated in *V*p shifts, particularly volume-regulated anion channels (VRACs; *e.g*., the leucine-rich repeat containing 8-isoform A; LRRC8A) (45, 246). TRPM2 is also activated by nicotinic acid dinucleotide phosphate (NAADP) (247), elevated upon GSIS (160, 285). Interestingly, TRPM2 was also reported to interact with peroxiredoxin 2, from which it can receive a redox signal (168, 186).

Action potential firing begins at [glucose] > 6 m*M* in mouse β-cells (49), stimulated by reaching a depolarization of up to −50 mV. Above −50 mV, Ca_V_ opening [predominantly Ca_L_ with minor contribution of R-, N-, and P/Q-type Ca^2+^ channels (228)] is intermittent with the opening of the voltage-dependent K^+^ channels (K_V_) in mice (150) or calcium-dependent K^+^ channels (K_Ca_) in humans (49) since K_V_ (K_Ca_) opening terminates Ca^2+^ entry, but their time-dependent deactivation allows a new 30–40 ms spike (210). Also, Na^+^ channels participate in upstrokes in a 30% β-cell population (289). Spikes return to a plateau *V*p of −50 to −40 mV, the level of which is also adjusted by the two-pore K^+^ channels TASK-1 and TALK-1 (50, 264). At 10 m*M* [glucose], periods of a high and low frequency of action potential spikes exist, including burst and silent interburst phases (210).

The latter is explained by a transient ATP consumption by sarco/ER-Ca^2+^-ATPase (SERCA) and plasma membrane Ca^2+^-ATPase (PMCA), that is, ATPases removing Ca^2+^ (252, 253). At >20 m*M* glucose, ATP synthesis is thought to overcome its consumption, leading to a permanent action potential firing (49), upon which 100% of K_ATP_ channels close (210). The amplitude becomes reduced by 15 mV after ∼3 min.

The resulting pulsatile Ca^2+^ entry elevates the cytosolic Ca^2+^ concentration [Ca^2+^]_c_. The accumulated Ca^2+^ pool acts simultaneously on the protein exocytotic machinery and thus stimulates the pulsatile Ca^2+^-dependent exocytosis of IGVs (213, 214, 260). In human PIs, the threshold is −60 mV, the frequency of action potential spikes is higher, whereas 5 ms spikes are grouped into shorter ∼2 s groups and their termination upon lowering glucose is slow (211). As for the [glucose] dependence in mice, 50% of K_ATP_ closing was already reported at 3 m*M*, keeping *V*p constant; while at 5 m*M* 93% and at 10 m*M* 97% of K_ATP_ channels were closed (251). Thus, *V*p depolarization is due to the closure of remaining ∼7% K_ATP_ in mouse PIs when [glucose] is increased above 5 m*M* (235).

In mouse β-cells, Ca_V_ isoforms Ca_V_1.2 and Ca_V_1.3 are responsible for 50%, Ca_V_2.1 for 15%, and Ca_V_2.3 for 25% of the whole-cell Ca^2+^ current, which is activated at −50 mV (228). Interestingly, R-type Ca_V_2.3 channels were reported to open exclusively during the second phase of GSIS (109). The protein kinase A (PKA) phosphorylation of Ca_V_1.2 and Ca_V_1.3 enhances their activity (122). Note that different groups of channels, not only Ca_V_, are involved in action potential spikes in different species, cultured cells or even within individual cells of PIs (210), which is outside the scope of this review.

The deactivation of Ca_V_ is switched predominantly by the opening of K_V_2.1 in rodents (150, 213) or K_V_2.2 and K_Ca_1.1 channels (BK channels) in humans (49, 101). A delayed rectifier K^+^ current is induced at positive *V*p down to −30 mV (215). The opening of K_V_2.1 channels repolarizes *V*p and thus closes Ca_V_ channels. The ablation of K_V_2.1 reduced Kv currents by ∼80% and prolonged the duration of the action potential, secreting more insulin. Mice with ablated K_V_2.1 exhibited lower fasting glycemia, but elevated insulin, and improved GSIS (100). Interestingly, glucose, glyceraldehydes, and 2-ketoisocaproate (KIC) were reported to increase Kv currents (284).

#### Ca^2+^ oscillations

Besides synergy with other channels, Ca_V_ opening intermittent with K_V_ opening leads to *V*p oscillations (action potential firing) (91), which induce primary oscillations in [Ca^2+^]_c_ (210). The latter is further modulated by a Ca^2+^ efflux from the ER (49, 280), lysosomes, IGVs, or mitochondria (see the Mitochondrial Ca^2+^ Signaling in Pancreatic β-Cells section). However, the ER Ca^2+^ efflux cannot be initiated without the preceding primary Ca_V_-mediated Ca^2+^ influx. The two components are superimposed, that is, fast cytosolic Ca^2+^ oscillations with 2–60 s periods and slow Ca^2+^ oscillations with periods reaching up to several minutes (15, 73). The resulting complex Ca^2+^ oscillations finally induce pulsatile insulin secretion. One can predict more IGVs to be secreted with a higher time-integrated cytosolic Ca^2+^concentration.

### Basic mitochondrial contribution to insulin secretion

#### ATP supply and its regulation in pancreatic β-cells

Undoubtedly, increasing ATP synthesis by OXPHOS with increasing [glucose] is the first prerequisite for GSIS (152, 156). OXPHOS respiration is determined as the oxygen consumption rate (OCR). The OCR of cultured β-cells or PIs, incubated with low (insulin nonstimulating) [glucose], increases after further glucose elevation (193). Simultaneously, mitochondrial IMM potential Δ*Ψ*_m_ also increases, indicating that the OCR increase is not due to uncoupling (protonophoric action), but stems from faster ATP synthesis, while the respiratory proton pumps are fully coupled *via* the protonmotive force (Δp = ΔΨ_m_ + ΔpH) to the H^+^ backflow through the ATP synthase.

OXPHOS can be semiquantified, when accounting for the ratio (*R*_r_) of OCR to OCR_Oligo_. OCR_Oligo_ values of nonphosphorylating respiration are set by oligomycin, blocking the ATP synthase, hence driven by the H^+^ leak. For rat INS-1E cells, the ratio *R*_r_ exhibits a sharp increase between 3 and 8 m*M* [glucose] with AC_50_ at ∼3.5 m*M* and saturation at >8 m*M* [glucose] in INS-1E cells (193). This AC_50_ roughly corresponds to the half-maxima of the surplus in the total cell ATP and in the insulin secretion rate (116). For human healthy and diabetic PIs, AC_50_ of 4.4 and 5.5 m*M* were found, respectively (47).

The parameter *A*_r_, where *A*_r_ = (OCR − OCR_Oligo_)/OCR_FCCP_, follows a very similar relationship to AC_50_ (193), reflecting the fraction of the maximum respiration (OCR_FCCP_) capacity used for ATP synthesis. The extent of this sharp increase in *R*_r_ or *A*_r_ perfectly correlates with the [glucose] range for which 50%–100% closure of the K_ATP_ ensemble proceeds, despite different ranges for rat *versus* mice *versus* human PIs (see The Synergy of Membrane Channels section). With oligomycin, the closure of the K_ATP_ ensemble in INS-1E cells is incomplete (194).

When a major fraction of ATP synthase molecules are incapable of synthesizing ATP in INS-1E cells, such as upon silencing of the subunit DAPIT, GSIS is virtually unchanged, although elevations of ATP were only ∼10% of those in nontransgenic cells (134). This interpretation stems from the reasoning that the second leg required for GSIS (*i.e*., redox signaling) was preserved, and the established lower ATP was able, together with H_2_O_2_, to close K_ATP_ (or simultaneously open TRPM2).

With isolated rat PIs, elevations from resting 2 m*M* up to insulin-stimulating 4 m*M* [ATP] (at 10 m*M* [glucose]) were found (43), while AC_50_ at ∼3 m*M* was reported for the total ATP rise and 50% K_ATP_ closure in mouse PIs, not correlating with AC_50_ of ∼12 m*M* for GSIS (210). Perhaps specific AC_50_ for the first phase should be considered. In α-toxin-permeabilized PIs, the 84% K_ATP_ closure occurred already at 1 m*M* ATP (251). The perifusion of human PIs with up to 7.5 m*M* [glucose] leads to ∼30% of maximum GSIS (267), with insulin release observed beginning at 3 m*M* (89). In humans, blood glycemia of 7.5 m*M* stimulates a fivefold increase in insulin (266). Note that there is no sudden increase in glucose after a meal in humans, instead glycemia increases over ∼30 min from ∼5 to 8 m*M* (61).

Description of the diabetic phenotype is out of scope in this review [but cf. Refs. (5, 17, 107, 248)]. Type 2 diabetes etiology originates not only from the impaired molecular mechanisms of insulin secretion but also from low-grade inflammation causing insulin resistance and promoting β-cell oxidative stress, ER stress, and cell death. Pancreatic β-cells first attempt to compensate the glucotoxic metabolic demand by enhancing their mass, which also elevates insulin production. Still, their exhaustion induces further pathogenesis, impaired β-cell biogenesis, leading to dedifferentiation and dysfunction (20). This further deteriorates insulin secretion. During the β-cell mass expansion phase of the type 2 diabetes development, the first GSIS phase is often missing, whereas the second phase is enhanced and prolonged, so higher time-integrated insulin release exists. This was therefore termed hyperinsulinemia (117).

#### Regulations of ATP synthase by ATPase inhibitory factor 1

**FIG. 3.**
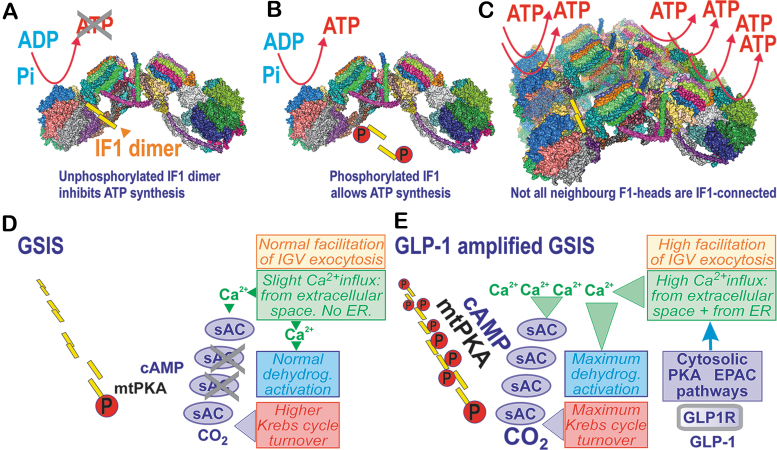
**IF1 adjusts glucose-sensing concentration range being hypothetically regulated by PKA upon GSIS and its amplification by GLP-1. (A, C)** Inhibitory binding of nonphosphorylated IF1 dimer within structure of ATP synthase dimer **(A)** and four adjacent ATP synthase dimers **(C)**, within model of crista segment. **(B)** Inability of phosphorylated IF1 to bind and inhibit ATP synthase. **(D)** Hypothetical phosphorylation of small fraction of IF1 molecules upon GSIS, when mild activation of the soluble adenylyl cyclase within mitochondrial matrix (mt-sAC) produces proper cAMP levels, required to adjust the accurate proper IF1 phosphorylation by the mitochondrial matrix PKA. Mt-sAC is activated by the increased CO_2_ due to a higher Krebs cycle (glucose metabolism) turnover upon GSIS and also the concomitant cytosolic Ca^2+^ oscillations relayed to the matrix cause oscillations in [Ca^2+^]_m_ superimposed onto the steady-state increasing [Ca^2+^]_m_ levels (cf. the Contribution of Mitochondrial Ca^2+^ to Insulin Secretion section). **(E)** Hypothetical higher IF1 phosphorylation state upon GLP-1 amplification of GSIS. Here, in addition to the situation described in **(D)**, the mt-sAC population could be more activated due to higher CO_2_, resulting from the additional activation of matrix dehydrogenases, which are superactivated (mt-sAC as well) by more integrally intensive [Ca^2+^]_m_ oscillations superimposed onto the steady-state increasing [Ca^2+^]_m_ levels. They are determined by cytosolic Ca^2+^ oscillations with a prolonged duration of bursts onto which the Ca^2+^ efflux from ER stores is also superimposed (cf. the Mitochondrial Ca^2+^ Homeostasis upon Receptor-Augmented Insulin Secretion section). As a result, hypothetically, a higher fraction of the matrix IF1 population should be phosphorylated and hence ATP synthesis could be more intensive. ER, endoplasmic reticulum; GLP-1, glucagon-like peptide 1; IF1, ATPase inhibitory factor 1; sAC, soluble adenylyl cyclase.

Searching for factors that adjust the [glucose] range to the sensing one (3–8 m*M* in INS-1E cells), we found ATPase inhibitory factor 1 (IF1) to be a key element (115, 116) (Fig. 3). This regulation adds to well-known settings of the sensing [glucose] range due to other factors, including the proper K_m_ of rodent glucose transporter GLUT2/SLC2A2 (human GLUT1/SLC2A1), K_m_, and the lack of product inhibition for glucokinase, existing smooth fluxes of glycolysis that supply the Krebs cycle, followed by the efficient supply of substrates (NADH, succinate) for the respiratory chain and OXPHOS (227, 290). Note that glucokinase is considered a glucose sensor. This notion is supported by its importance since inactivation of both glucokinase alleles leads to the maturity-onset diabetes of the young type 2 (MODY-2). Nevertheless, this causes defective K_ATP_ regulation (205).

IF1 was thought to be able to only inhibit the reverse mode of the ATP synthase, in which H^+^ ions are pumped into the intracristal space (ICS) across the c-subunit-ring of the membrane F_O_ moiety, whereas the energy is supplied by ATP hydrolysis to ADP ongoing at the F_1_ moiety. This is unlikely in primary cells; in cancer cells, this mode is mixed with the regular ATP synthesis (269).

However, evidence was found for the inhibition of ATP synthesis by IF1 *in vivo* (115, 116). A mild partial inhibition of a fraction of the ATP synthase (Fig. 3) may just set the proper glucose-sensing range in pancreatic β-cells. With silenced IF1 in INS-1E cells, the insulin secretion dependence on [glucose] was shifted far left with AC_50_ ∼1 m*M* (115). A similar shift with increasing [glucose] was observed for the glucose-induced surplus in total cell ATP, which was always higher in IF1-silenced cells (fivefold higher at 1 m*M*; about twice at 7 m*M* [glucose]), reflecting a mild inhibition of ATP synthesis in control cells. In contrast, the IF1 overexpression in INS-1E cells inhibited GSIS, so that the maximum saturated insulin release was about half, whereas AC_50_ was slightly right-shifted to 4.5 m*M* (116). A more profound shift to ∼7 m*M* was observed for the [glucose] dependence of cell surplus ATP levels, which were about halved at 1 m*M* and ∼25% at 7 m*M* glucose (116). These results reflected the ATP synthase inhibition *in vivo* by the excessive (overexpressed) IF1.

#### Structural aspects of IF1 interaction with ATP synthase *versus* cristae morphology

ATP synthase dimers are organized in arrays or rows along the crista rims (Fig. 3C), while actually determining the morphology of cristae. If we can approximate the two neighboring dimers by the revealed structure of the tetrameric porcine ATP synthase (80), we can also speculate on the actual IF1 localization *in vivo*.

The IF1 dimers bridge the two F_1_ moieties, however, not those within a single ATP synthase dimer, but between two neighboring dimers (Fig. 3A–C) (80). These connections (bridges) *via* the dimeric IF1 are lifted above the membrane of the crista edge. The membrane at this edge is bent into a sharp rim purely due to the single F_O_-dimer structure. IF1 is attached to the bottom of the interface between the α- and β-subunit, where it meets with the γ-subunit of the F_1_ moiety (74, 80). Speculatively, one may assume that both the F_1_ moieties bridged with the IF1 dimer cannot synthesize ATP. In this instance, not all dimers along the ATP synthase row of dimers could be connected by the IF1-IF1 bridges since if this was the case, no ATP synthesis could exist; all F_1_ moieties would be inhibited.

Moreover, the IF1 dimerization is prevented when IF1 is phosphorylated on Ser39 by PKA (68, 69). Also, fast degradation *via* the factor IEX1 was reported (230). Therefore, not only the regulation of IF1 expression *versus* degradation (53) but also PKA signaling provides fine-tuning of ATP synthesis. We hypothesize that the multifaceted natural regulation of IF1 and/or all ATP synthase subunits (including mtDNA-encoded) sets the proper activity within the ensemble of ATP synthases, which provides the properly adjusted rate of ATP synthesis in pancreatic β-cells. This complex regulation predetermines the glucose sensing that starts between 3 and 4 m*M* (strictly dependent on the elevated NOX4-redox signal) in INS-1E cells or isolated mouse PIs.

When the fraction of phosphorylated IF1 increases within the matrix, an even higher rate of ATP synthesis can be achieved, as simulated by IF1 silencing or additions of dibutyryl-cAMP, which increased cytosolic ATP levels (115). Also, GSIS was upregulated after the dibutyryl-cAMP treatment, but the upregulation ceased in IF1-knockdown cells, indicating that the IF1 phosphorylation enabling higher ATP synthesis was the important component of this mechanism. The dibutyryl-cAMP treatment also compensated the suppressing effect of IF1 on the cytosolic ATP and on the total released insulin amount (116).

#### Mitochondrial PKA pathways in pancreatic β-cells

PKA either phosphorylates suitable protein residues exposed to the cytosolic face of the outer mitochondrial membrane (OMM; PKA_OMM_) or even proteins of the mitochondrial matrix (mtPKA) (78, 292). The latter implies the existence of sensors leading to cAMP signaling in the matrix (187, 287). Thus, adenylyl cyclase mt-sAC (soluble adenylyl cyclase), phosphodiesterase mtPDE2A2 (2), and also mtPKA (286), were identified to be localized in the matrix. Indeed, the GPR receptor activator forskolin induced the phosphorylation of matrix proteins, such as IF1 (68, 69). cAMP cannot freely diffuse into the matrix, and no cAMP carrier is known (2); hence, the matrix cAMP pool is independent of the cytosolic one (44, 46). The ICS-localized or peripheral intermembrane space-localized PKA_IMS_ might phosphorylate the Complex IV COXIV-1 subunit, which prevents its inhibition by ATP and hence enhances respiration and OXPHOS (39). For PKA_IMS_, one could expect the cytosolic cAMP to penetrate at least to the peripheral intermembrane space.

Matrix mt-sACs are hypothetically activated by elevated matrix Ca^2+^, while experiments reported mt-sAC activation by bicarbonate, which increased matrix cAMP (34, 132). Nevertheless, no mtPKA activation under these conditions was found (132). Since CO_2_ is increasingly released when the Krebs cycle turnover is elevated, mt-sAC activation could occur upon the metabolic stimulation of insulin secretion. Similarly, increasing responses of matrix [Ca^2+^]_m_ to cytosolic Ca^2+^ oscillations and Ca^2+^ efflux from the ER (Fig. 3D, E) may activate the matrix mtPKA (3), the existence of which was found in *Drosophila* (286). Thus, OXPHOS is facilitated in the mitochondria of numerous tissues due to the Hsp70-mediated import of the NDUFS4 subunit of Complex I, initiated by phosphorylation, as well as by the phosphorylation of IF1 (69, 115, 116). The observed release of the PKA catalytic subunits by the increased ROS is also noteworthy (216, 243).

## Mitochondrial Ca^2+^ Signaling in Pancreatic β-Cells

### Contribution of mitochondrial Ca^2+^ to insulin secretion

#### Stimulation of matrix dehydrogenases and OXPHOS machinery by mitochondrial Ca^2+^

**FIG. 4.**
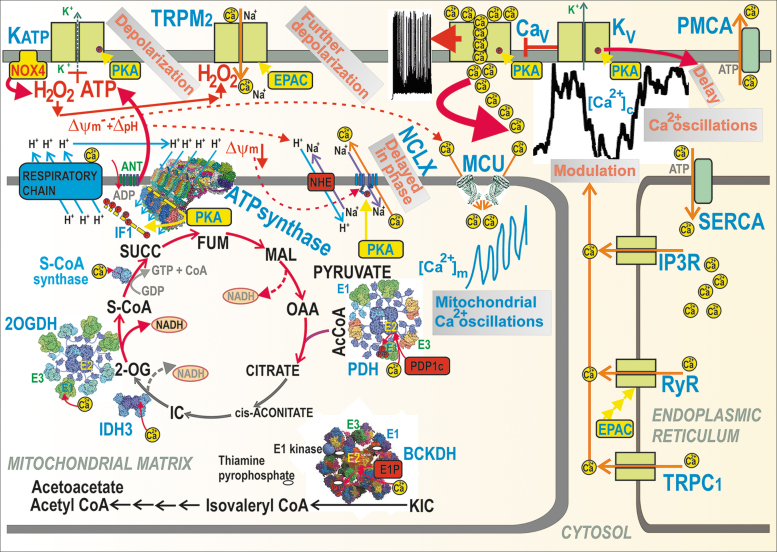
**Mitochondrial *versus* cytosolic Ca^2+^ oscillations and their ability to activate matrix dehydrogenases.** Action potential firing is reflected by cytosolic Ca^2+^ oscillations, which determine the steady-state increase in matrix [Ca^2+^]_m_ with superimposed [Ca^2+^]_m_ oscillations and concomitant activation of matrix dehydrogenases. Activation due to the PKA pathway is also indicated, demonstrating phosphorylation (*red circles*) of (i) IF1 hypothetically forming bridges (*yellow*) between the neighboring dimers of the ATP synthase within a *row* of dimers (four dimers are depicted with the indicated H^+^ backflow that leads to ATP synthesis); (ii) NCLX promoting activation *via* the Δ*Ψ*_m_ decrease; (iii) K_ATP_ channels (setting their sensing of ATP to ∼1 m*M* [ATP]); (iv) Ca_V_ channels, thus activating them. Similarly, the EPAC2A pathway (“EPAC”) reportedly activates TRPM2 and RyR. The *dashed arrows*, pointing to NADH, illustrate the sites where NAD^+^ is made from NADH due to pyruvate redox shuttles (Fig. 5). Ca_V_, voltage-dependent Ca^2+^ channels; EPAC, exchange proteins directly activated by cAMP; FUM, fumarate; IC, isocitrate; MAL, malate; NCLX, mitochondrial sodium calcium exchanger; RyR, ryanodine receptor; SUCC, succinate.

The stimulation of matrix dehydrogenases upon GSIS is one of the most plausible benefits provided by the Ca^2+^ influx into the matrix *via* the mitochondrial calcium uniporter (MCU) complex (41, 70, 71) (Fig. 4). The FAD-glycerol-3-phosphate dehydrogenase, localized on the outer IMM surface, is then instead influenced by the cytosolic Ca^2+^ penetrating into the intramembrane space or ICS (4, 165, 219, 252). Ca^2+^ activation was also reported for mt-sAC (34, 44, 46), which hypothetically leads to the phosphorylation of IF1 (69, 115, 116) by a putative matrix mtPKA (3, 132). mtPKA releases the IF1-mediated inhibition of the ATP synthase, thus enhancing ATP synthesis (69). A link to Ca^2+^ was suggested for the observation of 50% GSIS suppression upon ablation of the GTP-providing succinyl-CoA (S-CoA) synthetase, whereas the ablation of its ATP-providing form accelerated GSIS (126). We conclude that the mitochondrial Ca^2+^ transport represents a key factor of GSIS dependence on mitochondria.

#### Mitochondrial Ca^2+^ transport upon GSIS

The mitochondrial matrix content of bound Ca^2+^ and the free Ca^2+^ concentration [Ca^2+^]_m_ (124) are finely regulated by the Δ*Ψ*_m_-driven Ca^2+^ influx *via* the MCU complex (41), which is balanced by the Ca^2+^ influx, conducted by the Ca^2+^/2Na^+^ antiporter (mitochondrial sodium calcium exchanger [NCLX]) (38). The latter is driven by ΔpH *via* the Na^+^/H^+^ antiporter (plausibly NHE6/SLC9A6). Hypothetically, LETM1 may also ensure Ca^2+^/2H^+^ antiport, thus extruding Ca^2+^ from the matrix (202).

Mitochondrial Ca^2+^ participates in the first GSIS phase (70) and in GSIS potentiation by GLP-1 (71, 218). A sudden [glucose] elevation in primary β-cells induces the concomitant Ca_V_-dependent [Ca^2+^]_c_ oscillations, which are relayed to delayed steady-state increases in mitochondrial [Ca^2+^]_m_ up to saturation (252, 253). The observed [Ca^2+^]_m_ oscillations, superimposed onto the linearly increased [Ca^2+^]_m_, are roughly in phase with [Ca^2+^]_c_ oscillations. The higher the frequency of the action potential spike within a burst, the higher [Ca^2+^]_m_ amplitude was reached (252). These changes induced a biphasic increase in the ATP/ADP ratio with its second phase after 5 min (124, 252, 253).

The mechanism behind this is probably enabled by slightly retarded NCLX responses, in which Ca^2+^ influx exceeds the Ca^2+^ efflux during these transients and during the entire nearly linear [Ca^2+^]_m_ increase up to saturation. The major effect of such integrally elevated [Ca^2+^]_m_ is in the well-known Ca^2+^ activation of mitochondrial dehydrogenases (4, 164, 165, 219, 252) [doubted in Drews *et al*. (48)] (Fig. 4).

One cannot identify the above-described second phase in the ATP/ADP increase with the second GSIS phase, nevertheless in MCU-deficient β-cells, such a second-phase-ATP/ADP-increase was missing (252, 253). The [Ca^2+^]_m_ responses were slightly shifted up upon NCLX silencing (253). Insulin release from primary β-cells, monitored using Zn^2+^ as a surrogate, was stimulated either by high [glucose] independent of MCU deficiency (which led to delayed responses) or by K_ATP_ closing with tolbutamide, which ceased upon MCU deficiency (252). Hence, the activation of dehydrogenases was also delayed and was probably responsible for the observed second-phase ATP/ADP increase.

The overexpression of the Ca^2+^-binding protein S100G in the matrix of INS-1E cells prevented [Ca^2+^]_m_ increases responding to [Ca^2+^]_c_, blocked the glucose stimulation of respiration and ATP, thus reflecting the prevention of OXPHOS upon impaired [Ca^2+^]_m_ responses (271). Typical [Ca^2+^]_m_ elevations up to 880 n*M* dropped to 530 n*M*. In primary β-cells, S100G overexpression specifically attenuated the second GSIS phase, while the first phase did not decrease (271). This reflects a delay required for the full-extent activation of matrix dehydrogenases. In a more exaggerated way, this effect is also manifested during GSIS amplification by GLP-1 (90, 258).

Experiments suggested the essential requirement of MCU for GSIS in mice with an ablated MCU-pore, specifically in pancreatic β-cells (70). The insulin release was suppressed the first 5 min following the glucose administration, but after that, the time-integrated insulin release was equal to controls. Thus, in-phase MCU-mediated increases in [Ca^2+^]_m_ concomitant with [Ca^2+^]_c_ oscillations upon GSIS or GLP-1 amplification of GSIS (see the Mitochondrial Ca^2+^ Homeostasis upon Receptor-Augmented Insulin Secretion section) are among the precise mitochondrial machinery, which is required for optimum ATP synthesis.

The MCU complex is composed of the regulatory scaffolds MCU regulator 1 (MCUR1), the essential MCU regulator element (EMRE), and three isoforms of Ca^2+^-channel/sensors, termed mitochondrial calcium uptake proteins 1, 2, and 3 (MICU1,2,3) (127, 191). Mitochondrial Ca^2+^ transporters are well known to respond to Ca^2+^ released from the ER. This is reflected by the silencing of either MCU or MICU1, which reduced [Ca^2+^]_c_ oscillations and respiration rates and also decreased ATP production and GSIS (4). MCU was found to be activated by kaempferol (22).

A higher Δ*Ψ*_m_ allosterically blocks NCLX, hence Ca^2+^ efflux, and thus increases [Ca^2+^]_m_ (129). Mechanistically, this requires the interaction of Ser258 with positively charged residues of NCLX, which is disrupted by PKA phosphorylation, hence NCLX becomes insensitive to Δ*Ψ*_m_, and thus active. For pancreatic β-cells, this regulation implies a low NCLX activity at low [glucose] but high activity upon insulin-stimulating [glucose] (129). Speculatively, this allosteric effect may be behind the oscillation of [Ca^2+^]_m_ since each cycle of MCU-mediated Ca^2+^ influx may transiently or locally decrease Δ*Ψ*_m_, whereas the concomitant fraction of imported Ca^2+^ partially upregulates OXPHOS, hence adds to Δ*Ψ*_m_, which in turn would activate NCLX. The regulation of OXPHOS by cytosolic Ca^2+^ penetrating into the ICS probably occurs *via* the Ca^2+^-induced activation of Complex IV subunit Cox4.1, which disrupts its feedback inhibition by ATP (39, 114, 203). The impact on GSIS is yet to be studied.

#### Synchronization of cytosolic and mitochondrial Ca^2+^ upon GSIS

Within rodent islets, cooperation between β-cells exists. Synchronization of the electrical activity of the plasma membrane potential within the ensemble of cells in the islet results in synchronization of their cytosolic Ca^2+^ oscillations and other events (99, 111, 218). A few percent of pacemaker-like β-cells provides such synchronization. These cells were termed hub cells. Since it has been recognized that the second GSIS phase exists in PIs, but not in the β-cells isolated from islets, this cell cooperation was considered to substantiate the second phase. But, the delayed kinetics of the insulin granules (104) plus intercellular synchronization act in parallel.

Of course, major synchronization takes place within the individual β-cells. *At first*, the initial rise in ATP plus H_2_O_2_ upon elevating glucose sets the triggering event for Ca_V_ channels by closing K_ATP_, whereas H_2_O_2_ can hypothetically also activate TRPM2 channels. With other NSCCs or other synergic channels, depolarization reaches up to the *−*50 mV threshold of *V*p, ultimately activating action potential firing due to the intermittent opening of Ca_V_ channels and K_V_ channels.

*Second*, the pulsatile Ca^2+^ influx from the exterior causes *cytosolic Ca^2+^ oscillations*. Specifically at intermediate [glucose], such as ∼10 m*M*, Ca^2+^ oscillations might be terminated because of the transient exhaustion of cytosolic ATP by PMCA and SERCA (252, 253), thus creating silent interburst phases (210) [see the lag between burst [Ca^2+^]_c_ phases in Fig. 4—part of the records published in Plecita-Hlavata *et al*. (194)]. Moreover, under the activation of receptors, such as GPR (inositol-1,4,5-triphosphate receptor-diacylglycerol [IP3-DAG] signaling) or GLP-1 receptor (PKA and EPAC2 pathways), an additional amplifying Ca^2+^ efflux, is induced from the ER, *via* Ca^2+^ channels of TRPC1, ryanodine receptor (RyR), or IP3 receptor (IP3R). This ER Ca^2+^ efflux modulates and superimposes onto the existing cytosolic Ca^2+^ oscillations.

*Third*, cytosolic Ca^2+^ oscillations are relayed to the matrix, causing *oscillations in [Ca^2+^]_m_* superimposed onto the *steady-state increasing [Ca^2+^]_m_ levels* (252, 253). This is hypothetically allowed by the in-phase delayed Ca^2+^ efflux mediated by the NCLX Ca^2+^/Na^+^ antiporter, behind the instantly acting MCU. NCLX is inhibited by a higher Δ*Ψ*_m_, but due to higher ATP synthesis and concomitant H^+^ backflow *via* the ATP synthase c-ring, Δ*Ψ*_m_ is partially diminished, leading to NCLX activation (129). The Ca^2+^ uniport *via* MCU is driven by the Δ*Ψ*_m_ component of the protonmotive force Δp, whereas the electroneutral Ca^2+^/2Na^+^ antiport by NCLX is driven by the Na^+^-gradient, established by the Na^+^/H^+^ antiporter (NHE6), which is then driven by the ΔpH component of Δp.

*Fourth,* besides the matrix mt-sAC, the resulting *[Ca^2+^]_m_ elevation activates matrix dehydrogenases* (42, 164, 218) in the set pace, specifically: (i) the 8-MDa multienzyme complex of pyruvate dehydrogenase (PDH), in which Ca^2+^ binds to heterodimers of the E2 PDH subunit and the catalytic subunit of pyruvate dehydrogenase phosphatase (PDP1c) within the core of the complex, which leads to the PDP1-mediated dephosphorylation of E1 subunits. The PDH-complex contains a hollow core of numerous dihydrolipoate acetyltransferase subunits (E2), plus 12 E3-binding subunits (E3BP). E3BP attaches subunits of pyruvate decarboxylase (E1) and dihydrolipoate dehydrogenase (E3). Since the phosphorylated E1 causes the inhibition of the overall PDH reaction, the Ca^2+^-activated dephosphorylation is therefore the key event for the PDH activation (42).

(ii) BCKA-dehydrogenase (BCKDH) complex is activated by mechanism similar to (i), in which Ca^2+^ activation of the E1P-phosphatase occurs, which dephosphorylates the E1 subunit, which is otherwise inhibited by the phosphorylation enabled by the BCKDH-E1-kinase, and this is in turn inhibited by the cofactor thiaminepyrophosphate. Since the thiaminepyrophosphate-mediated kinase inhibition is strengthened by Ca^2+^, hence Ca^2+^ activates BCKDH (180). Moreover, there is a direct Ca^2+^-induced activation for (iii) Ca^2+^ binding to the E1 subunit of the 2-oxoglutarate dehydrogenase (2OGDH) multienzyme complex; (iv) Ca^2+^ binding to βγ-subunit interfaces of the hetero-octameric NAD^+^-dependent isocitrate dehydrogenase 3 (IDH3) (42), and (v) Ca^2+^ activates the GTP-producing S-CoA synthase by as yet unknown mechanism (126).

### Mitochondrial Ca^2+^ homeostasis upon receptor-augmented insulin secretion

#### Signaling by GLP-1 receptor for the amplification of GSIS

Produced in intestinal L-enterocytes, GLP-1 from the bloodstream activates its receptor (GLP1R) on the plasma membrane of pancreatic β-cells (170). GLP1R activation preferentially stimulates G-proteins Gαs, but also Gαq or Gα_11_ (Fig. 10), and recruits β-arrestin, depending on a biased agonism differently to different agonists, such as exendin-4 and oxyntomodulin (238, 275).

A scaffold protein β-arrestin promotes signaling *via* Gαs to cAMP, but also to CREB (238), extracellular regulated kinase ERK1,2 (169), and insulin receptor substrate 2 (IRS-2). This activates β-cell growth, differentiation, and β-cell identity maintenance (238). The major Gαs stimulation spreads signals *via* enhanced cAMP (65, 133, 177) and the initiation of PKA (143), plus the enhanced signaling *via* exchange proteins directly activated by cAMP 2A-isoform (EPAC2A) pathways (120). However, a putative cAMP-independent pathway may also exist at physiological 1–10 p*M* [GLP-1] (231). Prolonged cAMP production could even be induced by internalized GLP1R, which partly potentiates GSIS (255). *Ex vivo*, GLP-1 was found to act at a low range of stimulating [glucose] 6–7.5 m*M* (5–6 m*M* in isolated β-cells) (65, 133, 177, 231), which paradoxically is equivalent to fasting glycemia in mice.

The PKA pathway activation leads to a surplus [Ca^2+^]_c_ above that of the net GSIS (*i.e*., without any receptor stimulation) (151). This is achieved by the phosphorylation of K_V_ channels, leading to their deactivation. This prolongs the overall Ca^2+^-stimulation signals and induces somewhat lower frequencies of Ca^2+^ oscillations, but with each spike lasting longer (231, 265). Also, the second phase of GSIS might be potentiated by such mechanisms. Speculatively, the PKA-pathway evoked [Ca^2+^]_c_ surplus may activate Cox4.1 in the ICS if the concentration therein would reflect [Ca^2+^]_c_. PKA also phosphorylates snapin, a protein of the exocytotic machinery. This promotes soluble N-ethylmaleimide-sensitive factor (NSF) attachment protein receptor (SNARE)-complex formation by the interaction of synaptosomal nerve-associated protein 25 (SNAP-25) with synaptogamins of IGVs. Thus, exocytosis is facilitated within the first GSIS phase (236, 237).

A parallel EPAC2A pathway activation by the GLP1R signaling stimulates TRPM2 channels (285), providing the essential shift in depolarization in synergy with K_ATP_ and thus triggers the action potential firing. EPAC2A also enhances K_ATP_ closing (40, 75, 121). The EPAC2A pathway also promotes the docking and priming of IGVs by allowing Rab3A interaction with Rim2α (282); and a hypothetical interaction of EPAC2-Rim2α-Picollo trimers with Rab3A, again facilitating IGV exocytosis (254). Also, Ca^2+^-release is activated from the ER *via* the RyR-, which depends on Ca_V_ opening (120). A biased GLP1R stimulation *via* Gαq/11 may also stimulate Ca^2+^ release from the ER now *via* IP3R (23) and *via* TRPM4 and TRPM5 activation due to phosphorylation by protein kinase C (PKC) (231).

Experiments using simultaneous electrophysiological and Ca^2+^ oscillation monitoring (with Ca^2+^ fluorescent probes) found that at 2 m*M* glucose, but with 200 μ*M* tolbutamide blocking K_ATP_ (265), the GLP-1 analog liraglutide decreased the frequency of action potential spikes, which became individually wider. This reflects the PKA-mediated inactivation of Kv2.1 channels. With or without liraglutide, each action potential spike matched the triangular peak of the cytosolic Ca^2+^ rise. Its time-width increased from 2 s to about 5 s with liraglutide (265). The relative duration *versus* the active phase of Ca^2+^ spikes was ∼10% at 4 m*M*, ∼50% at 7 m*M*, and ∼80% at 9 m*M* glucose (59). Earlier experiments at 7.7 m*M* glucose and with GLP-1_(7-36)amide_ (preproglucagon_78-107_) also reported an increased duration of active and silent electrical activity (58).

Importantly, the observed delayed decay of Ca^2+^-responses is not only determined by the prolonged action potential spikes but is also affected by Ca^2+^, released from the intracellular stores, especially the ER, but also from mitochondria. The relay of these complex Ca^2+^-responses onto the in-phase intermittent responses of proteins of the exocytotic machinery (formation of SNARE complexes) and the resulting pulsatile IGV exocytosis were also monitored by surveying the ATP-activated currents conducted by the artificially overexpressed P2X2 cation channels (145). This was possible due to IGVs containing a high ATP concentration.

## Mitochondrial Metabolism of Pancreatic β-Cells

### Redox shuttles provided by several mitochondrial anion carriers

#### Redox shuttles

**FIG. 5.**
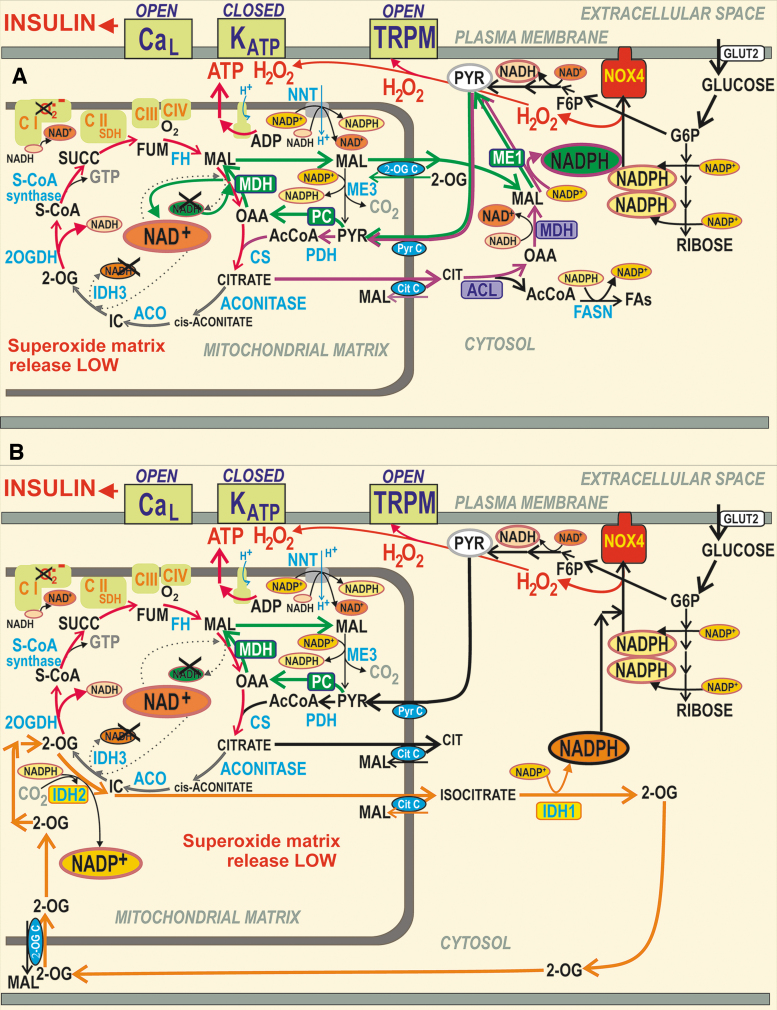
**Pyruvate-based redox shuttles transfer matrix NADH equivalents to elevate cytosolic NADPH. (A)** The *pyruvate/malate redox shuttle* (*green arrows*) and *pyruvate/citrate shuttle* (*violet arrows*). The pyruvate/malate redox shuttle bypasses PDH and the concomitant entry of the resulting acetyl-CoA into the Krebs cycle *via* the CS. This bypass exists due to the PC reaction producing OAA. Conditions upon glucose intake into pancreatic β-cells allow the reversed reaction of the matrix MDH2 that produces malate from oxaloacetate at the expense of NADH, which is converted to NAD^+^. That is why redox equivalents of NADH are transferred into cytosolic NADPH. The transfer is achieved by malate export *via* the “2-OGC” (SLC25A11) (183), where it is exchanged for 2OG, which is then imported to the matrix. The exported malate increases the cytosolic malate pool, which can be consumed by the ME1 reaction, driven by NADP^+^ and thus increasing the cytosolic NAPDH pool (82, 195). This reaction direction is driven by an instant return of pyruvate to the mitochondrial matrix ensured by the pyruvate carrier (MPC1 and MPC2, providing pyruvate-H^+^ symport). In this way, the cycle is achieved. The *pyruvate/citrate shuttle* is enabled by the citrate export from the matrix after the CS reaction (54). This truncated Krebs cycle has been confirmed using ^13^C-tracing, demonstrating that high amounts of the cytosolic citrate originate from glucose-derived acetyl-CoA (146). Citrate is exported by the citrate carrier (“Cit C”; SLC25A1), enabling citrate antiport with malate. Together with the pyruvate/malate redox shuttle, malate cycling occurs. The exported citrate is split in the cytosol by the ACL with CoA, yielding oxaloacetate and acetyl-CoA. The cytosolic isoform of MDH1 then converts oxaloacetate into malate, which is again used by ME1 to produce NADPH and pyruvate, which is finally imported back to the matrix. The ACL reaction and hence shuttle operation is minor compared with the acetoacetate pathway operating upon GSIS (52). Under low glucose conditions, levels of short-chain acyl-CoA are preserved. **(B)** The *pyruvate/isocitrate shuttle* (*orange arrows*) exists when the reductive carboxylation reaction of matrix IDH2 takes place. Unlike IDH3, which is the regular Krebs cycle enzyme providing NADH, IDH2 in a “forward” oxidative decarboxylation mode uses NADP^+^ plus citrate and produces NADPH and 2OG in the matrix. At high [glucose], such conditions are established instead to facilitate the reverse IDH2 reaction, which is the NADPH-driven reductive carboxylation of 2OG in the presence of CO_2_ (193). This is also facilitated by the Krebs cycle truncation, leaving a slow aconitase reaction and isocitrate formation so that the reverse IDH2 reaction occurs. The citrate carrier finally exports isocitrate to the cytosol, exchanging it for imported malate. The enhanced isocitrate pool in the β-cell cytosol is concomitantly consumed by the cytosolic IDH1, ensuring the NADP^+^-driven oxidative decarboxylation of isocitrate to 2OG, yielding NADPH (82). As a result, IDH1 within this shuttle contributes to another portion of the cytosolic NADPH increase upon GSIS (209). 2OG, as with the pyruvate/malate shuttle, is imported to the matrix, being exchanged for malate again by the oxoglutarate carrier. 2OG thus contributes to the mitochondrial matrix 2OG pool, consumed massively by the 2OGDH complex within the Krebs cycle. However, a portion of the matrix 2OG pool is used for another cycle of this shuttle, that is, for IDH2-mediated reductive carboxylation. 2OG, 2-oxoglutarate; 2OGC, 2-oxoglutarate carrier, mitochondrial; 2OGDH, 2-oxoglutarate dehydrogenase; ACL, ATP citrate lyase; ACO, aconitase; Cit C, citrate carrier, mitochondrial; CoA, coenzyme-A; CS, citrate synthase; F6P, fructose-6-phosphate; FASN, fatty acid synthase; FH, fumarate hydratase; IDH1, isocitrate dehydrogenase 1, cytosolic NADP^+^ dependent; IDH2, isocitrate dehydrogenase 2, mitochondrial NADP^+^ dependent; IDH3, isocitrate dehydrogenase 3, cytosolic NAD^+^ dependent; MDH, malate dehydrogenase; ME1, malic enzyme 1, cytosolic; OAA, oxaloacetate; PC, pyruvate carboxylase; PDH, pyruvate dehydrogenase; PyrC, pyruvate carrier, mitochondrial.

NADPH has long been considered to be a facilitator of GSIS (107, 110, 112, 183, 193, 209). However, the NADPH increase was not known to activate NOX4 upon GSIS, and PPP was thought to be inactive due to the product-inhibition of G6P-dehydrogenase (227, 290). Later, metabolomics confirmed a significant diversion of G6P flux to PPP upon GSIS (147, 240). Besides the two PPP enzymes, G6P-dehydrogenase and 6-phosphogluconate dehydrogenase, there is a contribution of three metabolic redox shuttles to the increasing [NADPH]_c_ upon GSIS (110), complying with the high pyruvate influx into the matrix (147). We recognize the pyruvate/malate, pyruvate/citrate, and pyruvate/isocitrate shuttle (Fig. 5).

Note also that metabolic pathways including metabolic shuttles are plastic, specifically when the key members are altered in their expression, such as identified upon glucotoxicity, lipotoxicity, and glucolipotoxicity in isolated human PIs (108). Therefore, the description below concerns with the most frequent schemes before and after glucose intake into intact pancreatic β-cells.

#### NADPH and NADH homeostasis in the cytosol and mitochondrial matrix upon GSIS

The redox shuttles, activated upon GSIS, do not allow maximum NADH to be produced in the mitochondrial matrix, but instead more NADPH is produced in the cell cytosol, representing a transfer of redox equivalents from the matrix to the cell cytosol (Fig. 5A, B). The shuttles concomitantly provide an independent, although minor NADPH source, supplying NOX4 to initiate redox signaling that enables insulin secretion upon elevated ATP. Mitochondrial malate dehydrogenase 2 (MDH2) produces less NADH, when compared with the situation of the 100% forward reaction, which proceeds at low [glucose].

Also, less NADH is produced, if isocitrate is not converted by the isocitrate dehydrogenase IDH3 to produce NADH due to the truncated Krebs cycle owing to the citrate export from the matrix, or when the concurrent reaction direction exists so that isocitrate dehydrogenase 2, mitochondrial NADP^+^ dependent (IDH2) is switched to the inverse (reductive carboxylation) reaction. The exported isocitrate promotes NADPH formation by the cytosolic isocitrate dehydrogenase 1, cytosolic NADP^+^ dependent (IDH1). Thus again, instead of one NADH molecule produced in the matrix, one NADPH molecule is formed in the cytosol.

Decreasing matrix NADH ([NADH]_m_) at high- *versus* low glucose conditions has one interesting consequence, a diminished matrix NADH/NAD^+^ ratio, which causes decreased superoxide formation, probably at the I_F_ flavin-site of Complex I (193). As a result, upon GSIS, we do have a dichotomic redox situation in the β-cell cytosol *versus* mitochondrial matrix. Whereas the cytosolic H_2_O_2_ elevation occurs due to NOX4 function, the matrix superoxide formation decreases (likewise H_2_O_2_ produced by superoxide dismutase MnSOD). Moreover, typical [NAD^+^]_m_, estimated, for example, in HeLa cells, is up to two orders of magnitude higher than [NADH]_m_. Values of 800 μ*M* [NAD^+^]_m_ and 5 μ*M* [NADH]_m_ were reported (33). During fast respiration upon GSIS, this difference actually leads to a situation in which each NADH molecule formed by the respective matrix dehydrogenases is instantly consumed by Complex I.

As for the matrix NADPH ([NADPH]_m_), we found that it decreases with increasing glucose. Specifically, the operation of the pyruvate/isocitrate shuttle and reductive carboxylation by IDH2 consumes NADPH significantly in INS-1E cells (193), and this is not balanced by the increased NADPH formation by the matrix malic enzyme 3 (ME3) nor by the increasing forward (Δp-consuming) mode of nicotinamide nucleotide transhydrogenase (NNT). The matrix ME3 forms pyruvate and NADPH from malate and NADP^+^ (85). The acute [NADPH]_m_ decrease could lead to a decrease in the reduced glutathione in the matrix, representing a resource sacrificed in exchange for the transfer of redox equivalents, ensuring elevations in cytosolic NADPH.

### Other regulators of redox homeostasis

#### Nicotinamide nucleotide translocase in pancreatic β-cells

**FIG. 6.**
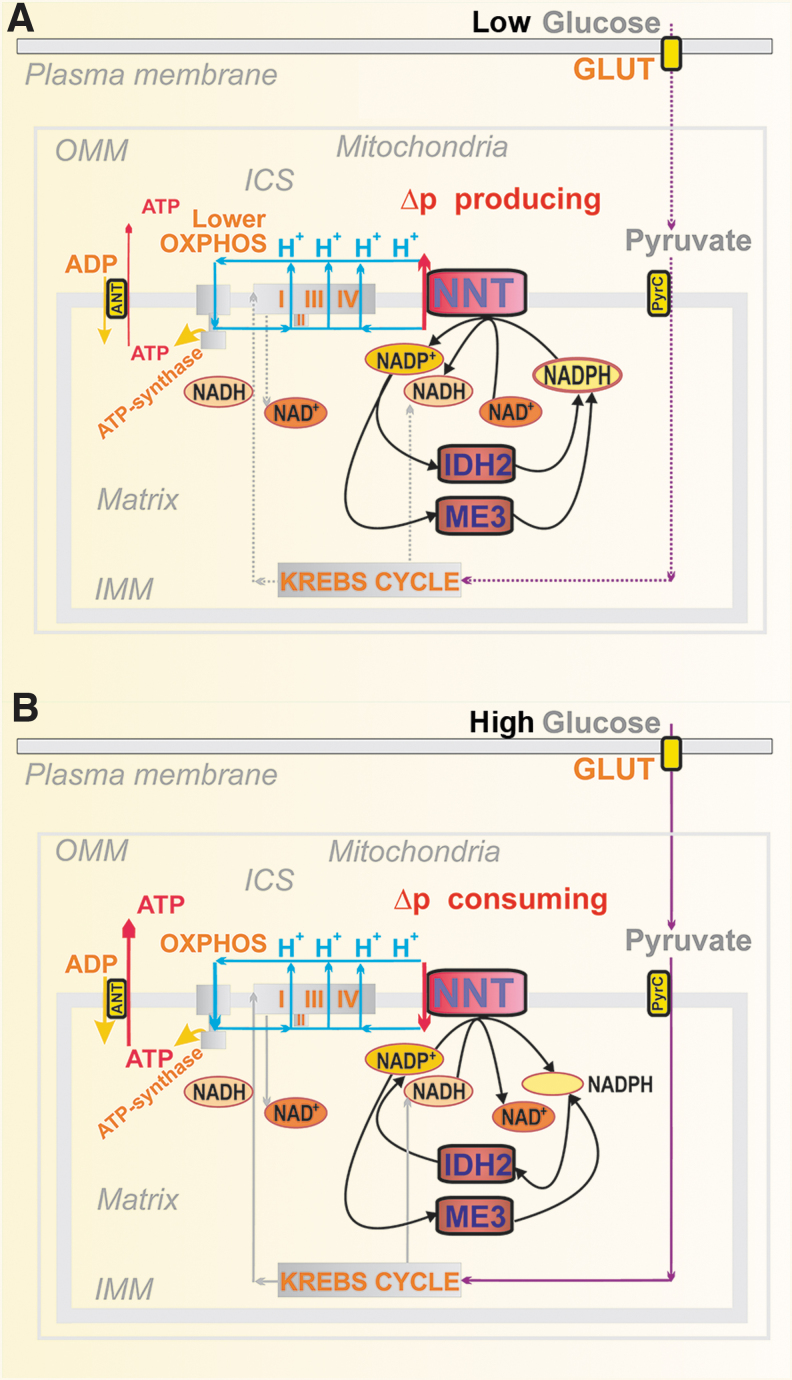
**Hypothetical reverse and forward mode of nicotinamide nucleotide translocase at low and high glucose, respectively. (A)** Low glucose conditions: hypothetical reverse NNT mode is depicted, when NNT acts as a proton pump, using the energy of NADPH and NAD^+^, converting them to NADP^+^ and NADH, respectively. It would be possible, since under low glucose conditions in pancreatic β-cells, a lower Δp would allow the proton pumping against that Δp. Possible NADPH sources could be the matrix IDH2 providing oxidative decarboxylation and ME3. **(B)** High glucose conditions: forward NNT mode is possible (193) when NNT uses Δp to translocate H^+^ into the matrix and drives NADPH formation from NADP^+^ with the simultaneous conversion of NADH to NAD^+^. This mode is highly probable upon GSIS since a high Δp is established, thus driving the H^+^ influx *via* NNT. Also, since the pyruvate/isocitrate shuttle is activated upon GSIS, it switches the IDH2 reaction direction to reductive carboxylation and NADP^+^ production. ME3, malic enzyme 3; NNT, nicotinamide nucleotide transhydrogenase.

Contradictory findings were reported for the mitochondrial NNT in PIs (Fig. 6). This IMM enzyme exposes its active site to the mitochondrial matrix. In a thermodynamically favored forward mode upon GSIS, NNT consumes Δp (Fig. 6B) by allowing H^+^ import into the matrix, tightly coupled with the conversion of NADP^+^ to NADPH and with the simultaneous NADH conversion to NAD^+^ (220). In this forward mode, NNT contributes to the matrix [NADPH]_m_ pool. Since NNT acts downstream of the redox shuttles, it cannot alter or affect them. Nevertheless, if all these shuttles operate, than IDH2 consumes NADPH and since ME3 cannot balance this consumption, matrix NADPH decreases upon GSIS (193).

Despite we could not indicate the reverse mode at low [glucose] (193), NNT was reported to function in the reverse mode (225), in which it pumps protons and thus provides a Δp surplus. This should be coupled with the consumption of NADPH and NAD^+^, yielding NADP^+^ and NADH. Such a mode would be possible, since at low [glucose] respiration and ATP synthesis exhibit somewhat slower rates, establishing a lower Δp, than with high [glucose]. The H^+^ pumping against an intermediate Δp would be possible at rather high NADPH/NADP^+^ ratio. In contrast, at high [glucose], NNT acts against the higher Δp. However, no direct observation of the actual H^+^ flux direction was conducted (225). By comparing Δ*Ψ*_m_, monitored using fluorescent probes, we demonstrated Δ*Ψ*_m_-increases in NNT-silenced INS-1E cells, supporting the existence of the forward NNT mode, which produces NADPH (193).

Neither experiments relying on the comparison of C57BL/6J *versus* C57BL6/N mice were conclusive. They reported that an in-frame five-exon deletion in the *Nnt* gene spontaneously occurred in C57BL/6J mice, thus removing exons 7–11, causing a complete absence of NNT protein (62, 63, 257). The C57BL6/J mouse strain was claimed to have a highly suppressed GSIS. However, other studies normally used knockouts backcrossed into the C57BL6/J-mice background as controls for GSIS and it exhibited high insulin secretion rates [*e.g*., Plecita-Hlavata *et al*. (194) and Wong *et al*. (273)]. The discrepancy originates from the fact that initially only the quantitative trait loci were identified. Thus, a mere correlation with deletions in the *Nnt* gene was assumed, and verifications using the artificial *Nnt* expression can be regarded as inconclusive, since the *Nnt* expression *per se* could enhance insulin secretion. This could be subsequently interpreted as an apparent GSIS suppression in C57BL6/J mice (273).

#### Malate/aspartate shuttle in low and high glucose conditions

**FIG. 7.**
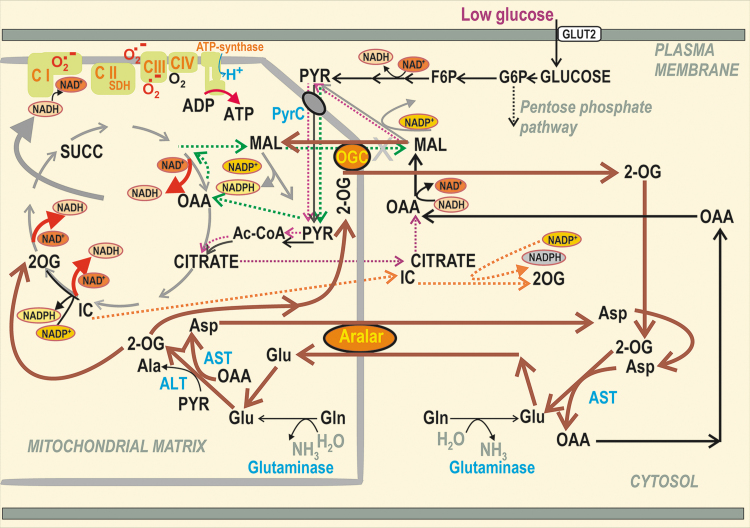
**MAS is plausible when pyruvate-based redox shuttles are not operating.** The MAS (*brown*) could participate in metabolic fluxes in pancreatic β-cells at low nonstimulating [glucose], when the pyruvate-based redox shuttles do not provide the opposite malate fluxes for the 2OGC. Moreover, the MAS transfers redox equivalents of NADH into the mitochondrial matrix; however, matrix NADH was found to decrease upon GSIS and relies on at least one of the two aspartate–glutamate antiporters, that is, SLC25A12/AGC1/aralar (18, 217) and SLC25A13/AGC2 (193) (data not shown). The key enzymes are alanine aminotransferases (cytosolic ALT1 and mitochondrial ALT2; also termed glutamate pyruvate transaminases, GPT1 and GPT2). Within MAS, ALT2 catalyzes the conversion of pyruvate plus l-glutamate to 2OG and l-alanine, whereas ALT1 (omitted for simplicity) would catalyze the reaction in the reverse mode. Analogously, there are aspartate aminotransferases, cytosolic AST1, and mitochondrial AST2 (also termed glutamate oxaloacetate transaminases, GOT1 and GOT2). In MAS, AST2 converts oxaloacetate plus l-glutamate to 2OG and l-aspartate, whereas AST1 should catalyze the opposite reaction to complete the cycle. Due to the reverse character of the aminotransferase reaction, its direction depends on the glutamate metabolism. AGC1, aspartate–glutamate antiporter SLC25A12 (Aralar); ALT, alanine aminotransferase; Aralar, aspartate–glutamate antiporter SLC25A12 (AGC1); AST, aspartate aminotransferase (aka glutamate oxaloacetate transaminase, GOT); GPT, glutamate pyruvate transaminase; MAS, malate/aspartate shuttle.

The malate/aspartate shuttle (MAS) was assumed to play a significant role in pancreatic β-cells (147, 240). However, interpretation of the relevant metabolomics data must be provided with caution since they mostly do not resolve metabolites of the mitochondrial matrix *versus* those from the cytosolic compartment (which is typically greater). One must consider that the metabolite transport direction within the active MAS is the opposite of the pyruvate redox shuttles (193) (Fig. 7). Their existence documented by numerous experiments over the last two decades (107, 110, 112, 183, 193, 209) thus excludes metabolite fluxes required for MAS operation at high [glucose].

The 2-oxoglutarate carrier (2OGC) mediates the malate efflux coupled with the 2-oxoglutarate (2OG) uptake to the matrix at high [glucose], whereas the malate import coupled with the 2OG export is required for MAS, if it exists. Unlike with pyruvate-based redox shuttles, at least one of two glutamate-aspartate antiporter isoforms is required for MAS, enabling the glutamate import in exchange for aspartate export from the matrix. In contrast, the aspartate needs to be imported as a part of the pyruvate/malate redox shuttle. However, glutamate formed in the matrix was suggested to be exported to the β-cell cytosol to facilitate IGV maturation and exocytosis (30, 72, 92, 93, 153, 155, 250). This would again require the opposite direction of glutamate flux.

At low [glucose], both aspartate–glutamate antiporters can participate in MAS, that is, SLC25A12/AGC1/aralar (18, 217) and SLC25A13/AGC2 (193). The existence of MAS was derived from the essential requirement of transaminases (aminotransferases) and aspartate–glutamate antiporters for β-cells (18, 217). Metabolomics studies evidenced a decrease in total cell aspartate at the initiation of GSIS, while aconitate, citrate, isocitrate, malate, or fumarate instantly rose, and elevations of 2OG and succinate were delayed until 15 min (241). Elevations in metabolites originate from the disbalance between producing *versus* consuming reactions, while the latter is slower; whereas for losses of metabolite, the producing reactions are slower. Hence, the observed aspartate losses reflect this disbalance.

Due to providing cytosolic glutamate, MAS was implicated in the GLP-1 amplification of GSIS, but not in GSIS itself (72). The ablation of cytosolic transaminase AST1/GOT1 reversibly transforming 2OG and aspartate to oxaloacetate plus l-glutamate led to the lack of GLP-1 effects. Further experiments are required to evaluate whether the three pyruvate-redox shuttles operate and interfere or not with MAS upon the GLP-1 amplification of GSIS, notably in the sustained second phase.

#### β-Hydroxybutyrate dehydrogenase and acetoacetate metabolism

**FIG. 8.**
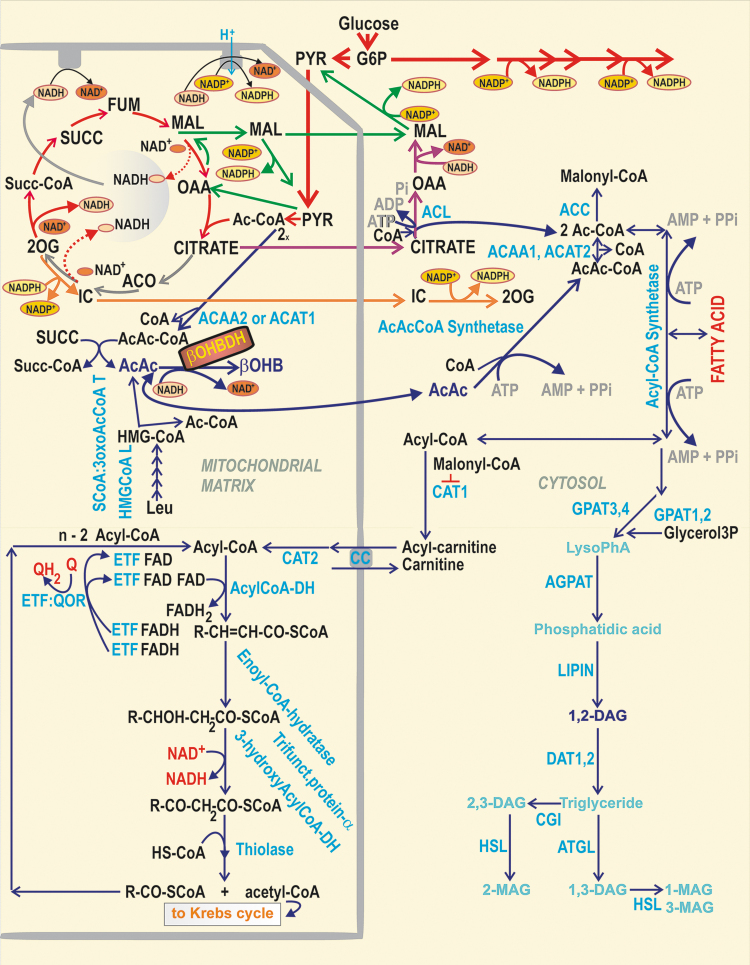
**β-OHB formation and FA metabolism in pancreatic β-cells.** The scheme describes three selected metabolic branches: (i) β-OHB *formation* and its relationship to leucine metabolism; (ii) *FA β-oxidation*; and (iii) the cytosolic *glycerol/FA cycle*. As for (i), at high [glucose], succinate is interconverted with AcAcCoA to S-CoA and acetoacetate by SCoA:3oxoAcCoAT (52). As part of leucine metabolism during a series of oxidative reactions (“β-like oxidation”) resembling FA β-oxidation, HMG-CoA is split by HMGCoAL into acetyl-CoA and acetoacetate. Besides being converted by β-OHBDH, acetoacetate can escape to the cytosol. Distinct enzyme isoforms convert two molecules of acetyl-CoA into CoA and AcAcCoA in the mitochondrial matrix. The latter are ACAT1 and ACAA2, whereas in the cytosol, there are ACAT2 and ACAA1. Cytosolic acetyl-CoA was suggested to facilitate the acetylation of proteins, which might speculatively enhance GSIS (189, 190). (ii) *FA β-oxidation*: FA is imported *via* CD36 into β-cells, where AcylCoA-synthetase (ACSL), localized externally to the ER membrane and OMM, converts FAs to acyl-CoAs, whereas the cytosolic CAT1 (synonymous for carnitine palmitoyltransferase, CPT1) converts acyl-CoAs to acylcarnitines (207). The carnitine carrier (SLC25A20) provides the import of acylcarnitines into the matrix, exchanging them for carnitine. The matrix CAT2/CPT2 converts acyl carnitines to acyl-CoAs. The following chain of reactions, termed FA β-oxidation, shortens the FA-acyl chain by two carbons, involving acyl-CoA dehydrogenases, enoyl-CoA hydratase, 3-hydroxyacyl-CoA dehydrogenase, and β-thiolase. The product of a single cycle is just acyl-CoA shortened by two carbons plus acetyl-CoA. The FA β-oxidation is regulated *via* the inhibition of CAT1/CPT1 by malonyl-CoA, formed by ACC from acetyl-CoA. (iii) *Cytosolic glycerol/FA cycle* (197): elevated glucose is converted to glycerol3P, which is esterified by acyl-CoAs by GPATs (bound externally to ER and OMM) to LysoPhA. The latter is further esterified by AGPAT (bound to ER) to PhA. At the ER surface or lipid droplets, lipins transform PhA to 1,2-DAG, initiating PKC signaling and activating Munc13-1. DAG is also acylated there to TG, by diacylglycerol *O*-acyltransferase-1 and -2 (DGATs). Simultaneously, the lipolytic branch is provided by the cytosolic ATGL, hydrolyzing TG to DAG, upon the facilitation of perilipin (data not shown) and CGI-58 protein (CGI) on the lipid droplet surface. DAG is hydrolyzed to MAG by HSL, again facilitated by perilipin. The created MAGs can overactivate the GPR119 receptor (Fig. 10). The glycerol/FA cycle is completed by the hydrolysis of MAG to glycerol and FAs by the plasma membrane-associated ABHD6 (data not shown), whereas glycerol is exported from β-cells. β-OHB, β-hydroxybutyrate; β-OHBDH, β-hydroxybutyrate dehydrogenase; ABHD6, alpha/beta-hydrolase domain containing 6, monoacylglycerol lipase; ACAA, acetyl-CoA acyltransferase; AcAcCoA, acetoacetyl-CoA; ACAT, acetyl-CoA acetyltransferase; ACC, acetyl-CoA carboxylase; ACSL, long-chain acyl-CoA synthetase; AGPAT, 1-acylglycerol-3-phosphate acyltransferase; ATGL, adipose triglyceride lipase; CAT, carnitine acyltransferase; CGI, comparative gene identification 58, ATGL co-activator (aka ABDH5); CPT, carnitine palmitoyltransferase; DAG, diacylglycerol; DAT, DGAT, diacylglycerol *O*-acyltransferase; FA, fatty acid; glycerol3P, glycerol-3-phosphate; GPAT1,2, glycerol-3-phosphate acyltransferase 1,2; GPAT3,4, glycerol-3-phosphate acyltransferase 3,4 (1-acylglycerol-3-phosphate O-acyltransferase); GPR, G-protein-coupled receptor; HMG-CoA, hydroxymethyl-glutaryl-CoA; HMGCoAL, hydroxymethyl-glutaryl-CoA lyase; HSL, hormone-sensitive lipase; LysoPhA, lysophosphatidic acid; MAG, monoacylglycerol; OMM, outer mitochondrial membrane; PhA, phosphatidic acid; PKC, protein kinase C; SCoA:3oxoAcCoAT, succinyl-CoA:3-ketoacid-CoA transferase; Succ-CoA, S-CoA, i.e. succinyl-CoA; TG, triglyceride.

β-Hydroxybutyrate dehydrogenase (β-OHBDH) is exclusive to the matrix in rodent pancreatic β-cells, playing an important role in redox homeostasis (149, 178). In pioneering investigations with hepatocytes, the β-OHBDH reaction was suggested to precisely reflect the matrix NAD^+^/NADH ratio, which would therefore determine the ratio of (total) β-hydroxybutyrate/acetoacetate concentration (178). However, since the estimated order of magnitude for the matrix NAD^+^/NADH ratio is >100, such an excess of β-hydroxybutyrate is unlikely. Since we reported the increase in this ratio upon GSIS (193), one could speculate that also matrix β-hydroxybutyrate rises upon GSIS (Fig. 8). However, acetoacetate can also be exported to the cytosol, where it is utilized by other reactions (149, 178). This was thought to facilitate insulin secretion *via* the formation of various acyl-CoA derivatives (Fig. 8) (149), which could acetylate proteins thus speculatively enhancing GSIS (189, 190). β-Hydroxybutyrate (https://www.brenda-enzymes.org/enzyme.php?ecno=1.1.1.30) can also be formed in the cytosol of human β-cells.

Long-chain acyl-CoAs were also reported to bind to the KIR6.2 subunit of K_ATP_ (28), which potently activates this channel (27, 77). Since upon GSIS, there is a reduction in total cell acyl-CoAs and malonyl-CoA (146, 198), such a reduction could facilitate K_ATP_ closure (146). Alternatively, FA β-oxidation (long-chain acyl-CoA shortening) could also provide the redox signaling toward K_ATP_ or TRPM2 (79, 123, 223), as with KIC (194) (see the Mitochondrial Contribution to Insulin Secretion Stimulated by BCKAs and FAs section).

#### Phosphoenolpyruvate cycle and role of pyruvate kinases

Another cycle, the phosphoenolpyruvate (PEP) cycle was suggested to act in the low glucose conditions. The PEP cycle is cataplerotic, beginning by the mitochondrial PEP-carboxykinase 2 (PEPCK2) conversion of oxaloacetate to PEP, which is exported by the citrate carrier (SLC25A1) from mitochondria. Cytosolic pyruvate kinases (PKs, isoforms constituent M1, recruitable M2 and L), existing in beta cells (167) use then the cytosolic PEP to convert it to pyruvate, which is coupled to ATP formation from ADP. Pyruvate enters mitochondria, where is metabolized either by PDH or by pyruvate carboxylase (PC). The PC flux completes the cycle by pyruvate conversion to oxaloacetate.

Pyruvate kinase isoform recruitable M2 (PKM2) and pyruvate kinase isoform L (PKL), allosterically activated by fructose 1,6-bisphosphate, were recently reported to aid K_ATP_ closure, as derived from patch-clamp experiments in excision mode combined with PK activation by a small-molecule activator (141). The authors exemplified PEP cycle switched on/off in the β-cell responses to intermediate 9 m*M* glucose, when *V*p and or [Ca2+]c bursts phases are interchanged with the interburst phases (Fig. 4). The decreased cytosolic ATP/ADP ratio was explained on the basis of the PEP cycle providing ATP synthesis by PK, that is, by “substrate” phosphorylation of ADP, independent of OXPHOS. Naturally low ATP sets K_ATP_-channels open, which occurs before glucose elevation and/or after termination of the burst phase at 9 m*M* glucose. When OXPHOS continues to elevate ATP further, PEP cycle is less active and more importantly the Krebs cycle control strength overcomes that of PEP cycle. Consequently, the burst phase begins at 9 m*M* glucose. Also, PKM2 and PKL activator failed to improve GSIS in PEPCK2-knockout mice (1).

### Glutamine and glutamate in pancreatic β-cells

#### Glutamine and glutamate metabolism

The reaction direction of mitochondrial glutamate dehydrogenase (GDH) in pancreatic β-cells was thought to favor the provision of glutamate and NAD^+^, while consuming 2OG, ammonium, and NADH (30, 92, 153, 155, 250). This would also contribute to decreasing [NADH]_m_, if acting upon GSIS. During fasting, GDH is activated by ADP and leucine, while at high [glucose], GDH is inhibited by GTP and ATP (67, 278).

Glutamate is exported from the matrix by the glutamate carrier GC1 (SLC25A22) (30, 92, 155). Also, pyruvate utilization by aminotransferases has a certain impact, despite being minor compared with the utilization by the matrix PDH complex and by the oxaloacetate anaplerosis provided by pyruvate carboxylase (6). Thus, cytosolic alanine aminotransferase (ALT1) and mitochondrial ALT2 [also termed glutamate pyruvate transaminases, GPT1 and GPT2 (188)] could catalyze the conversion of pyruvate plus l-glutamate to 2OG and l-alanine (279), which diminishes the matrix glutamate pool. The reverse GPT2 reaction would then produce glutamate and add pyruvate to its fast-consuming pool. Alternatively, AST1/GOT1 and mitochondrial AST2/GOT2 reversibly convert oxaloacetate plus l-glutamate to 2OG and aspartate.

Glutamine is also utilized in PIs, reportedly promoting leucine-stimulated insulin secretion at low [glucose], when phosphate-dependent glutaminase produces glutamate, which is further oxidized by GDH (67).

#### Glutamate regulation of insulin secretion

Glutamate was first suggested to facilitate GSIS (30, 92, 154, 155, 229), which was questioned by subsequent reports (24, 148). The glutamate effects are instead connected with IGV biology (214, 260). The specific uptake of glutamate into IGVs was found, being driven by ΔpH_IGV_, established on the IGV membranes by the V-ATPase (10). Anion influx, namely Cl^−^ influx by the ClC3 transporter, also helps to build ΔpH_IGV_. Additions of membrane-permeant dimethyl glutamate were reported to amplify both phases of GSIS, increasing the frequency of insulin granules merging with the plasma membrane (72).

Sufficient glutamate content inside the IGV lumen results from its uptake mediated by glutamate transporters VGLUT1,2,3 balanced by the EAAT2-mediated glutamate efflux (66). The ablation of VGLUTs reduced the GLP-1-induced amplification of GSIS but not GSIS itself (72). Ablation of the plasma membrane sodium-coupled neutral amino acid transporter 5 (SNAT5) also led to a reduced GLP-1 amplification of GSIS (86).

## Mitochondrial Contribution to Insulin Secretion Stimulated by BCKAs and FAs

### Insulin secretion by BCKAs involves mitochondrial retrograde redox signaling

#### Branched-chain amino acids *versus* BCKAs

A mixed meal leads to elevated levels of amino acids in circulation (244). During fasting or numerous pathologies, branched-chain amino acids (BCAAs) and BCKAs are also elevated in plasma. We will only discuss situations when pancreatic β-cells sense BCAAs and BCKAs in the islet microcirculation, or when these compounds are formed by metabolism. Thus, BCKAs, KIC (leucine metabolite) (7), 2-ketoisovalerate (KIV; valine metabolite), and 2-ketoisomethylvalerate (KMV; isoleucine metabolite) stimulate profound insulin secretion at low glucose (83, 88, 138–140, 163, 189, 190). Leucine can also exert effects by allosterically activating GDH (278).

**FIG. 9.**
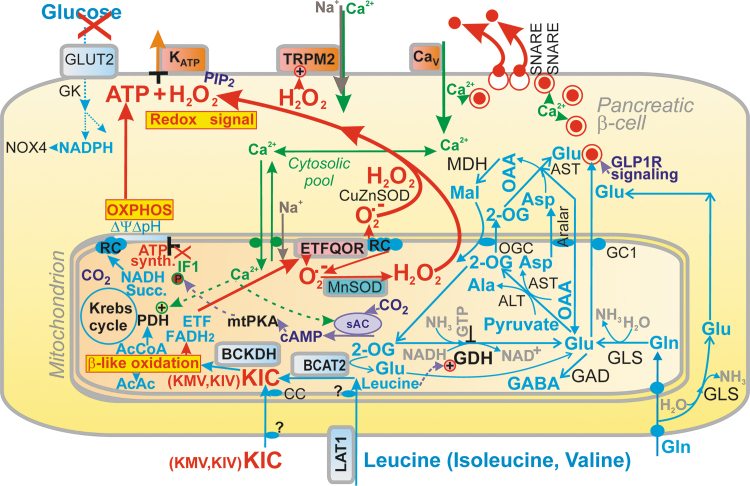
**Mitochondrial retrograde redox signaling determines insulin secretion by BCKAs.** KIC, KMV, and KIV may arise from blood capillaries (imported into the matrix *via* the carnitine carrier, CC) or by being converted from leucine, isoleucine, and valine, respectively, by the branched-chain aminotransferase reaction in the mitochondrial matrix (BCAT2). The BCAA import is ensured by the plasma membrane LAT1 transporter. The BCKDH, which provides electrons to ETF, exists exclusively in β-cell mitochondria and initiates a series of reactions of β-like oxidation. ETF:QOR accepts electrons from two ETFs, when it converts Q to QH_2_, and as side reactions produces superoxide together with its elevated production at sites I_Q_ and III_Qo_ (cf. Fig. 11). In the mitochondrial matrix, superoxide is transformed to H_2_O_2_ by MnSOD, but by CuZnSOD in the intermembrane space and cytosol. The elevated mitochondrial/cytosolic H_2_O_2_ substantiates the retrograde redox signaling that targets K_ATP_ to ensure its closure together with ATP (and possibly also TRPM2). As a result, Ca_V_ channels are open, providing the Ca^2+^-signal for IGV exocytosis. The end products of the β-like oxidation of KIC contribute to OXPHOS. Thus, AcCoA enters the Krebs cycle to form citrate, whereas AcAc can be partly exported to the cytosol or converted to β-OHB by a reverse reaction of β-OHBDH at the expense of NADH (Fig. 8). A link to glutamate and glutamine metabolism is also indicated, including the MAS (Fig. 7), which may exist together with BCKA-stimulated insulin secretion at low glucose, since at low glucose, the pyruvate-based redox shuttles are not highly active. AcAc, acetoacetate; AcCoA, acetyl-CoA; BCAA, branched-chain amino acid; BCAT2, branched/chain amino acid transferase 2, mitochondrial; BCKA, branched-chain ketoacid; BCKDH, branched-chain ketoacid dehydrogenase; ETF, electron transfer flavoprotein; ETFQOR or ETF:QOR, electron transfer flavoprotein:quinone oxidoreductase; GAD, glutamate decarboxylase; GC1, glutamate carrier; GLS, glutaminase; IGV, insulin granule vesicle; KIC, 2-ketoisocaproate; KIV, 2-ketoisovalerate; KMV, 2-ketoisomethylvalerate; OXPHOS, oxidative phosphorylation; Q, ubiquinone; QH_2_, ubiquinol.

Retrograde redox signaling (*i.e*., from mitochondria to cell cytosol, nuclei, or other organelles) is provided when these BCKAs are oxidized in mitochondria (29). This is substantiated by the elevated mitochondrial superoxide formation due to BCKA oxidation, whereas superoxide is transformed to H_2_O_2_ either by the matrix MnSOD or by the intermembrane space CuZnSOD (Fig. 9).

Despite there being details missing on how BCAA or BCKA are transported to the mitochondrial matrix, a well-known exclusively matrix BCKDH complex converts CoA plus BCKAs to proper BCKA-CoAs, that is, to isovaleryl-CoA, isobutyryl-CoA, and methyl-isobutyryl-CoA from KIC, KIV, and KMV, respectively. The entire series of reactions, a β-like oxidation (resembling FA β-oxidation) continues, for example, for KIC *via* methylcrotonyl-CoA carboxylase (MCC), methyl-glutoconyl-CoA hydratase (MGCoAH), and 3-hydroxy-3-methylglutaryl-CoA lyase (HMGCoAL), leading to the end products acetyl-CoA, which drives the Krebs cycle, and acetoacetate.

#### Mechanism of superoxide formation upon oxidation of BCKAs

The BCKDH reaction includes FAD as an electron acceptor. Subsequently, two electron transfer flavoprotein (ETF) molecules reoxidize the resulting FADH_2_ since each ETF is only a single-electron carrier. ETFs otherwise transfer electrons from 11 different mitochondrial flavoprotein dehydrogenases to the IMM ubiquinone (Q) pool. During BCKA oxidation, the transfer of electrons proceeds from one electron-reduced ETF one at a time to the lower potential FAD center of the electron transfer flavoprotein:quinone oxidoreductase (ETFQOR) (268). One electron is transferred to the iron cluster. ETF:QOR thus accepts two electrons, and this is coupled with the reduction of ubiquinone to ubiquinol (Q to QH_2_) with a transient semiubiquinone formation (268). This is a clue to the enhanced superoxide formation upon BCKA oxidation.

Since QH_2_ binds to the Complex I Q-binding site, the excessive supply of QH_2_ by ETF:QOR causes a feedback inhibition of the ongoing Q reduction to QH_2_ in Complex I. As a result, mitochondrial superoxide formation is accelerated at the so-called I_Q_ site of superoxide formation (Fig. 11). The second possible source could be due to the ETF:QOR reaction itself, while electrons are leaking from flavin to oxygen at site E_F_ (Fig. 11), forming initially a radical pair and finally superoxide (25). The third possible source is given by the excessive acetyl-CoA (propionyl-CoA) entry or methylmalonyl and S-CoA entry into the Krebs cycle. Since such enhanced catabolism accelerates respiration, an enhanced superoxide formation can be expected at Complex I site I_F_ and site I_Q_, and Complex III site III_Qo_, when the capacity of electron transfer is exceeded. Also, as discussed above, acetoacetate influences the established redox homeostasis.

#### Insulin secretion due to BCKAs

Insulin secretion upon elevated BCKA depends on increases in cytosolic ATP plus H_2_O_2_, now both supplied by mitochondria (Fig. 9). Indeed, BCKDH silencing blocked KIC-stimulated insulin secretion, as did the mitochondrial-matrix-targeted antioxidant SkQ1 (194). In contrast, the transaminase inhibitor aminooxyacetate had no effect. The blockage correlated with the suppressed matrix superoxide release, which was otherwise elevated by KIC. So, the H_2_O_2_ diffusion from the matrix substantiates the retrograde redox signaling from mitochondria. We admit the KIC doses may be supraphysiological in these experiments. A magnitude of a minimum threshold should be further investigated, which still creates sufficient mitochondrial superoxide/H_2_O_2_ *in vivo* to substantiate redox signaling reaching the plasma membrane and concomitant insulin exocytosis.

The most plausible terminal targets are located in the plasma membrane. They could be Cys residues of K_ATP_, or an oxidizable Met residue of TRPM2 (223), or both. Note that TRPM2 or other NSCCs or chloride channels are essentially required to shift the depolarization to the −50 mV threshold required for the opening of Ca_V_, and so for action potential firing (119, 221). This −50 mV threshold cannot be achieved without NSCCs or Cl^−^ channels, even when 100% of K_ATP_ channels are closed. The latter is undoubtedly established by a highly elevated cytosolic ATP resulting from β-like oxidation and concomitant OXPHOS.

### Redox signaling from mitochondria upon stimulation of insulin secretion by long-chain FAs

#### Fatty acid-stimulated insulin secretion requires mitochondrial β-oxidation and signaling *via* the GPR40 receptor

In rodent and human physiology, a mixed or fatty meal leads to the intestinal formation of chylomicrons that are brought by the circulation system to PIs after a few hours in humans, while also lipolysis is inhibited by secreted insulin (61). In contrast, the lipid- or FA-mediated secretion of GLP-1 by intestinal L-enterocytes comes earlier (173). Thus, a fatty meal induces insulin secretion *via* GLP-1 endocrine effects on PIs, whereas the further delayed insulin secretion due to chylomicrons arises at a time when even the 1-h-long second GSIS phase is terminated. So not only due to molecular mechanistic reasons, but due to physiological timing, it is crucial to study fatty acid-stimulated insulin secretion (FASIS).

**FIG. 10.**
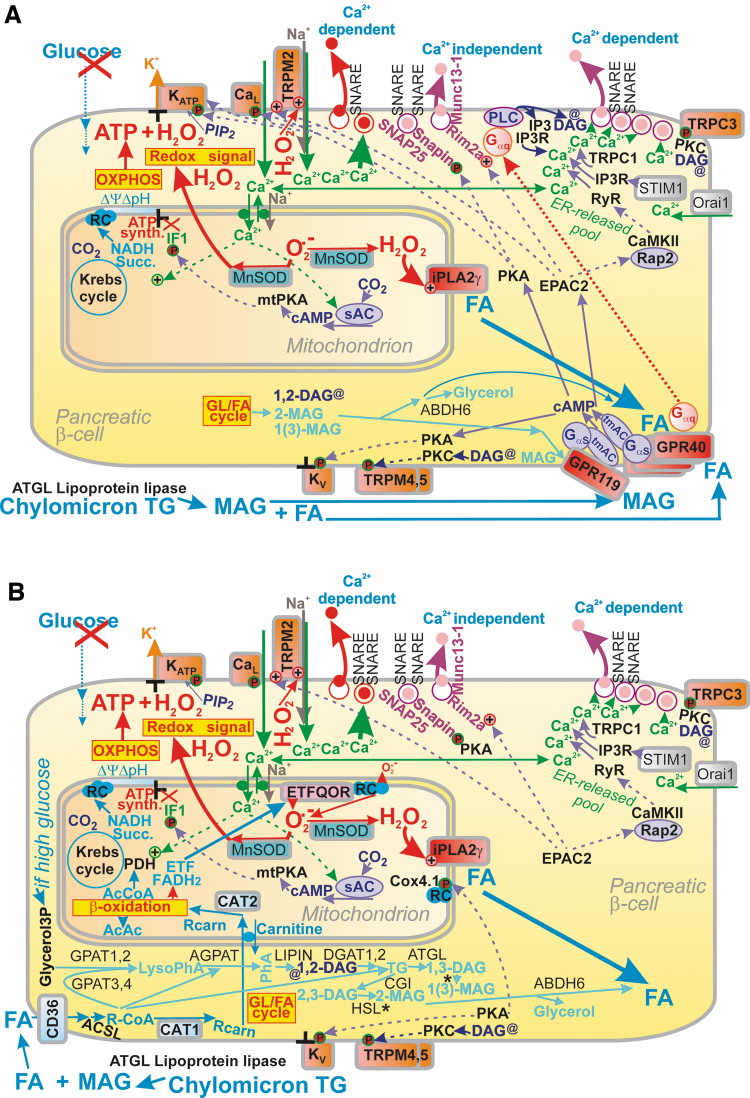
**Receptor and metabolic pathways determining FASIS. (A)** Receptor component of stimulation is emphasized. **(B)** Metabolic component is emphasized. When present in islets capillaries, for example, in incoming chylomicrons, FAs are cleaved by lipoprotein lipase (ATGL being the most specific β-cell isoform) and may either **(A)** *act via receptor pathways* to stimulate the GPR40 metabotropic receptor (94), in parallel with the second product of the cleavage, MAG, which signals *via* GPR119. Alternatively, **(B)** *the metabolic pathway begins* by FA import into the cell by the CD36 transporter and by ACSL conversion to acyl-CoAs. As for **(A**), the activation of both receptors leads to Ca_V_-mediated action potential spikes and concomitant pulsatile insulin secretion. GPR40 acts *via* the Gαq/11, thus activating PLC, which leads to IP3 and DAG release (for DAG downstream pathway, see @). IP3 activates additional Ca^2+^ efflux from the ER *via* the IP3R, which is initiated either by the preceding Ca_V_ opening (95) or by PLC-TRPC-induced Ca^2+^ efflux from the ER (276). The most prominent pathway downstream of DAG involves the PKC-mediated phosphorylation of TRPM4 and TRPM5 to activate them. As a result, together with TRPM2, activated by Ca^2+^ and H_2_O_2_, these channels strengthen the necessary shift to −50 mV depolarization at the 100% closed K_ATP_ ensemble. The K_ATP_ closure is ensured by the metabolic component of FASIS **(B)**. The two components are mutually interrelated since the canonical GPR119 signaling and the biased GPR40 signaling leads to the cAMP-mediated activation of the PKA and EPAC2 pathways (65, 187, 287). PKA phosphorylates the Ca_V_β2 subunit to activate it, phosphorylates K_ATP_ (see legend of Fig. 2) and inhibits Kv channels, which prolongs the already more intensive Ca^2+^ influx (179). Snapin, which allows IGV docking to the plasma membrane, is also PKA-phosphorylated, enabling initiation of the snapin SNARE complex with a lipid-anchored protein, the SNAP-25 (236). The EPAC2 pathway is based on its guanine nucleotide exchange activity. This induces further TRPM2 activation (285), regulates K_ATP_ (121) plus priming of the interaction of Rim2a with Munc13-1, required for the syntaxin 1 interaction of IGV [which activates IGV exocytosis (282)] and, finally, the activation of RyR- mediated Ca^2+^ efflux from the ER (120). FAs imported by CD36 are converted to acyl-CoAs by AcylCoA-synthetase (ACSL), whereas CAT1 converts acyl-CoAs to acyl-carnitines (207). The carnitine carrier (SLC25A20) imports acylcarnitines into the matrix, exchanging them for carnitine. The matrix CAT2 converts acyl carnitines to acyl-CoAs, which is followed by FA β-oxidation (see also Fig. 8). As described in the Mechanisms of Insulin Secretion section, all the benefits of activation also occur for mitochondrial metabolism, that is, activations upon GSIS and its receptor-mediated amplification (cf. Fig. 4). Also, similar redox signaling due to the increased superoxide formation upon FA β-oxidation occurs during the metabolic branch of FASIS, as with BCKA-stimulated insulin secretion (cf. Fig. 9). Elevated ATP from OXPHOS fortified by FA β-oxidation and elevated cytosolic H_2_O_2_ due to the increased H_2_O_2_-release from the matrix close the K_ATP_ channel (possibly also TRPM2), as they do upon GSIS. Overactivation of GPR40: pathways **(A, B)** are also interconnected because of the intramitochondrial redox signaling [elevated matrix superoxide/H_2_O_2_ due to FA β-oxidation (Fig. 11) directly activates mitochondrial phospholipase iPLA2γ/PNPLA8 (98, 103, 105)]. The phospholipase iPLA2γ cleaves both saturated and unsaturated FAs from the phospholipids of mitochondrial membranes. The cleaved free FAs diffuse up to the plasma membrane, where they activate GPR40 (103). FASIS in iPLA2γ-knockout mice or its isolated islets yields ∼30% insulin in the first fast phase of insulin secretion compared with wt mice (Holendová *et al.*, unpublished data). This supports the existence of such an acute mechanism *in vivo*. Overactivation of GPR119: FASIS in the presence of high [glucose] (which by itself would stimulate GSIS) also involves the so-called glycerol/FA cycle combining simultaneous lipogenesis and lipolysis, as suggested by Prentki *et al.* (197). Enzymes involved in this cycle are described in the legend of Figure 8. An important intermediate of the glycerol/FA cycle is 1,2-DAG, which initiates PKC signaling (and TRPM4,5 activation) and activates Munc13-1 to facilitate IGV exocytosis. Moreover, created MAGs (* indicates its diffusion toward GPR119) can diffuse to the plasma membrane and overactivate the GPR119 receptor there. ABDH6, alpha/beta-hydrolase domain containing 6, monoacylglycerol lipase; CaMKII, Ca^2+^/calmodulin-dependent protein kinase II; FASIS, fatty acid-stimulated insulin secretion; IP3, inositol-1,4,5-triphosphate; IP3R, inositol-1,4,5-triphosphate receptor; iPLA2γ, Ca^2+^-independent phospholipase A2 isoform γ; Orai1, calcium release-activated calcium modulator 1; PLC, phospholipase C; Rap2, Ras-related protein 2; RC, respiratory chain; SNAP-25, synaptosomal nerve-associated protein 25; SNARE, soluble N-ethylmaleimide-sensitive factor (NSF) attachment protein receptor; tmAC, transmembrane adenylyl cyclase; TRPC, transient receptor potential canonical.

*In vivo*, there is always a concomitant parallel portion of insulin secretion stimulated by 2-monoacylglycerol (MAG), cleaved from postprandial chylomicrons in PI capillaries by lipoprotein lipase (Fig. 10) (37, 158, 182, 272). The resulting MAG and long-chain FAs (37, 158, 182, 272) stimulate their own receptors (171). Adipose triglyceride lipase (ATGL) is the major isoform that cleaves triglycerides in PI capillaries (192), besides secretory phospholipases A2 (64). MAG activates metabotropic receptor GPR119, providing signaling *via* Gαs and cAMP (76, 87, 94, 96, 157, 171). It has been questioned whether the levels of FAs bound to albumin can initiate FASIS (102, 261), but this could happen with metabolic syndrome and/or obesity and type 2 diabetes since circulating FA levels are then elevated.

The consensus view was that sufficient glucose must always be present for FAs to induce any insulin secretion (51, 76, 87, 94–96, 157, 206, 245, 276). However, FASIS was described to exist at low (insulin nonstimulating) [glucose] (32, 103, 106, 194), but not at zero glucose (32). Human islets perifused without glucose did not increase respiration with long-chain FAs, but released insulin (32), whereas both increasing respiration and insulin release were observed with long-chain FAs plus 5.5 m*M* glucose. FASIS was evidenced by FA triggering an action potential with a prolonged duration at low (insulin nonstimulating) [glucose] (57) and concomitantly increased Ca^2+^ influx and hence potentiation of insulin secretion (55, 184). As a result, FASIS should be based on the metabolic components plus metabotropic receptor GPR40 signaling (76, 87, 94, 96, 103, 106, 130, 157, 201, 222, 256, 281, 288). It has to be established whether these two branches are mutually independent.

The major receptor pathway of FASIS (Fig. 10A) includes the metabotropic receptor GPR40, as evidenced by its ablation or its point R258W mutation since both impaired FASIS (222). The major pathway downstream of GPR40 initiates signaling *via* the Gαq/Gα11 (226), activating phospholipase C-β- (PLC-β-) mediated hydrolysis of phosphatidylinositol 4,5-bisphosphate into DAG and IP3 (23). The major axis involves the phosphorylation of TRPM4 (TRPM5) channels by PKC activated by DAG (231) or TRPC3 activation (276). As with TRPM2, the opening of TRPM4, TRPM5, or TRPC3 determines the necessary depolarization shift, despite the 100% closed K_ATP_ ensemble. The K_ATP_ closure is provided by the metabolic component of FASIS, that is, by FA β-oxidation providing ATP and H_2_O_2_, which may also function under low glucose conditions.

Signaling along the axis of GPR40-Gαq/Gq11-PLC-IP3 promotes additional Ca^2+^ efflux from the ER (initiated by the Ca_V_ opening, Fig. 4) *via* the IP3R, forming a Ca^2+^ channel of ER membranes (13, 64). Alternatively, a synergy exists for the plasma membrane channel calcium release-activated calcium modulator 1 (Orai1) with IP3R1 and stromal interaction molecule 1 (STIM1), sensing ER Ca^2+^ (259). If biased GPR40-Gαs signaling occurs, also the EPAC2-RyR route of Ca^2+^-release from ER might contribute to Ca^2+^ oscillations. GPR40 also initiates pathways of protein kinase D (PKD), activated by DAG (56), signal-regulated kinase 1 and 2 (ERK1/2) (201), and p21-activated kinase 4 (PAK4) (21). The latter regulates cytoskeletal dynamics, facilitating IGV exocytosis. Signaling downstream of GPR40 slightly increases respiration during 1-h incubations (130). Thus, PKC (224) and downstream ERK1/2 signaling stimulates OXPHOS, hence mitochondrial ATP synthesis (224).

Long-chain FAs are also imported into pancreatic β-cells by the sirtuin-activated CD36 FA transporter (125) (Fig. 10B). If short-chain FAs are present, they act *via* the GPR41 metabotropic receptor and contribute to the fine-tuning of insulin secretion in both fed and fasting states (200, 263). Similarly, the metabotropic receptor GPR120, having a different selectivity for agonists, mediates the amplification and/or stimulation of insulin secretion, by, for example, α-linolenic acid and polyunsaturated FAs (12, 172).

#### FA synthesis *versus* β-oxidation

During GSIS, due to the operation of pyruvate/isocitrate and pyruvate/citrate shuttles, conditions are set for FA synthesis. Metabolomics studies confirmed this, while observing an increase in free palmitic acid after transitions from low to high [glucose] (240). FA metabolism is even considered to be a prerequisite for GSIS since GSIS attenuation was observed in isolated PIs with inhibited triglyceride lipolysis (162, 176), in mice with deleted lipase specifically in β-cells (60) or in ATGL-knockout mice (192). But the net FASIS was not affected by the ATGL deletion. In contrast, at low [glucose] FA β-oxidation readily proceeds in pancreatic β-cells, supplying OXPHOS (Figs. 8 and 10B). Fifty to seventy percent of FAs generated by long-chain acyl-CoA synthetase (ACSL) are recycled into lipogenesis (197).

#### Retrograde redox signaling upon FASIS

**FIG. 11.**
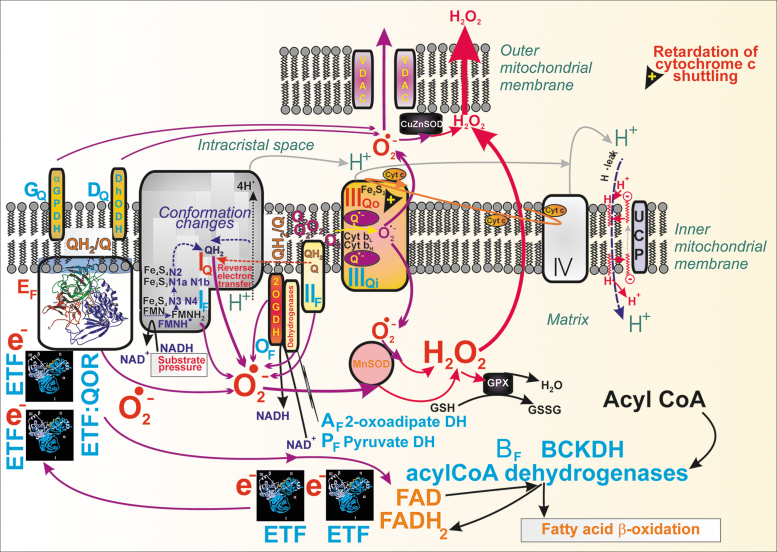
**Superoxide formation due to FA β-oxidation or oxidation of BCKAs.** An overview of locations for mitochondrial superoxide sources (*blue capitalized fonts*), termed according to the nomenclature introduced by Brand (25) is shown with those which increase superoxide formation upon FA β-oxidation or the β-like oxidation of BCKAs emphasized (in *red*). The electron-transfer flavoprotein:ubiquinone oxidoreductase (ETFQOR) is a common key electron transfer link between the initial dehydrogenases of these reactions and the respiratory chain complexes III and IV (29, 268). ETFQOR accepts electrons sequentially from two ETFs as single-electron carriers, while converting ubiquinone (Q) from its IMM pool to QH_2_. The electron leak from flavin to the oxygen leads to a radical pair formation and subsequent superoxide formation within the ETF:QOR itself (site E_F_). Moreover, the requirement of ETF:QOR to react with Q effectively outcompetes Q as the Complex I substrate, resulting in relative electron transfer retardation over the whole respiratory chain, hence superoxide is formed at its sites I_Q_ and III_Qo_. Finally, due to the increasing acetyl-CoA entry (propionyl-CoA entry for KIV; through methylmalonyl and succinyl-CoA) and NADH entry into the Krebs cycle, the excessive formation of superoxide at site I_F_ may also contribute. After the conversion of superoxide to H_2_O_2_ by the matrix MnSOD and the intermembrane space CuZnSOD, the ongoing H_2_O_2_ efflux from mitochondria can be regarded as redox signaling. As in other types of mitochondria, there are in total six sites acting at the ∼280 mV redox potential of the NADH/NAD^+^ isopotential pool (index F, flavin) and five sites acting at the ∼20 mV redox potential of the QH_2_/Q isopotential pool (index Q) (25). Of these, only the superoxide sources with more intensive production at higher Δ*Ψ*_m_ are attenuated by uncoupling proteins (103). This also involves the reverse electron transfer to the Complex I site I_Q_. In turn, superoxide formation at site IF increases with the increasing NADH/NAD^+^ ratio (“substrate pressure”). When cytochrome c shuttling (*orange elliptic arrow*) is retarded, then the Complex III site III_Qo_ provides major superoxide formation, which cannot be attenuated by uncoupling. IMM, inner mitochondrial membrane.

FA β-oxidation is based on mitochondrial flavoprotein dehydrogenases, such as the short-chain acyl-CoA dehydrogenase (EC 1.3.8.1), medium-chain acyl-CoA dehydrogenase (EC 1.3.8.7), long-chain acyl-CoA dehydrogenase (EC 1.3.8.8), and very long-chain acyl-CoA dehydrogenase (EC 1.3.8.9). All of them donate electrons to ETF:QOR *via* ETF and therefore contribute to the mitochondrial superoxide formation, which after conversion to H_2_O_2_ serves as redox signaling (Fig. 11). Therefore, the mechanism is similar as for the β-like oxidation of BCKAs. Even at low [glucose], the ETF-ETF:QOR redox relay to Complex I and III of the mitochondrial respiratory chain serves as the electron acceptor for dehydrogenases of β-oxidation. Interestingly, GPR40 signaling also activated NOX2 (181).

#### Redox-sensitive mitochondrial phospholipase iPLA2γ amplifies FASIS

Notably, the first FASIS phase (the second FASIS phase only moderately) was highly amplified by the action of mitochondrial phospholipase iPLA2γ (Ca^2+^-independent phospholipase A2 isoform γ [PNPLA8]), providing the cleaved mitochondrial FAs to GPR40 in insulinoma INS-1E cells (98, 103) and in mouse islets (Holendová *et al.*, unpublished data). The iPLA2γ is directly activated by H_2_O_2_, and it is also activated by the intramitochondrial redox signaling resulting from FA β-oxidation (103). The activated iPLA2γ cleaves free long-chain FAs from mitochondrial phospholipids. The cleaved long-chain FAs diffuse to the plasma membrane and subsequently stimulate GPR40 (Fig. 10A, B).

This was indicated by the direct observation of FA diffusion to the plasma membrane using the FA-sensitive fluorescent protein ADIFAB (103). Thus, the pool of free long-chain FAs generated by the glycerol/FA cycle (Figs. 8 and 10B) and CD36-mediated import is enriched by FAs cleaved from mitochondrial membranes. In this way, FAs self-accelerate the GPR40 signaling and hence insulin secretion. In INS-1E cells, ∼66% of the FASIS first phase was dependent on GPR40, and nearly the same 66% of it was blocked upon the silencing of iPLA2γ (or its ablation in mice, unpublished data). Hypothetically, the remaining part may depend on the elevation of ATP plus redox signaling from β-oxidation.

#### Mixed GSIS and FASIS

At high [glucose], triglyceride synthesis alternates with triglyceride hydrolysis in pancreatic β-cells (197). Because of ATP consumption, this cycling is futile. Since DAG is one of the intermediates of this cycle, it may provide all the above-specified signaling (Figs. 8 and 10B). Moreover, free FAs released during the cycle enrich the net GSIS with this supplemental FASIS. Indeed, the addition of FAs to β-cells and PIs at insulin-stimulating [glucose] amplifies GSIS, whereas β-cell acyl-CoA levels increase and appear to rapidly esterify glycerol-3-phosphate into lysophosphatidic acid and several different glycerolipids (51). This partly replenishes cytosolic NAD^+^. Also, glycerol-3-phosphatase produces glycerol and thus regulates glycolysis, the cellular redox state, ATP production, and other important branches of metabolism (175). The largest amount of insulin was secreted by INS-1E cells when palmitic acid plus 25 m*M* glucose were added, relative to either palmitic acid stimulation alone (∼80% of maximum) and GSIS alone (∼30% of maximum) (103).

Higher glucose decreases acyl-CoA levels in pancreatic β-cells (146). Previously, acyl-CoAs were suggested to activate K_ATP_ (26), hence declining acyl-CoAs would ease the K_ATP_ closure. Moreover, the metabolism of the remaining long-chain acyl-CoAs leads to superoxide/H_2_O_2_ formation, which also aids the opening of Ca_V_. In contrast, incoming higher glucose levels in pancreatic β-cells increase malonyl-CoA (81, 146), which inhibits carnitine palmitoyltransferase 1 (CPT1) and hence FA β-oxidation. This in turn opens the way for FA synthesis stemming from the ATP citrate lyase (ACL) reaction after the citrate efflux from the mitochondria (174). Nevertheless, the silencing of ACL and FA synthase in β-cells did not affect GSIS (113).

Upon increasing [glucose], glycerol-3-phosphate is also esterified, so the abundance of long-chain saturated monoacylglycerols increases (174, 291). These MAGs additionally stimulate insulin secretion *via* the GPR119 receptor and downstream PKA and EPAC2 pathway; the latter notably facilitates IGV priming by activating the protein Munc13-1 (291) (Figs. 8 and 10A). Note that this is similar to the GPR40 activation by the FAs cleaved in the cell interior, that is, from the mitochondrial membranes by mitochondrial phospholipase iPLA2γ.

## Abbreviations Used

2OG: 2-oxoglutarate
2OGC: 2-oxoglutarate carrier, mitochondrial
2OGDH: 2-oxoglutarate dehydrogenase
β-OHB: β-hydroxybutyrate
β-OHBDH: β-hydroxybutyrate dehydrogenase
ABHD6: alpha/beta-hydrolase domain containing 6, monoacylglycerol lipase
ACAA: acetyl-CoA acyltransferase
AcAc: acetoacetate
AcAcCoA: acetoacetyl-CoA
ACAT: acetyl-CoA acetyltransferase
ACC: acetyl-CoA carboxylase
AcCoA: acetyl-CoA
ACL: ATP citrate lyase
ACO: aconitase
ACSL: long-chain acyl-CoA synthetase
AGC1: aspartate–glutamate antiporter SLC25A12 (Aralar)
AGPAT: 1-acylglycerol-3-phosphate acyltransferase
ALT: alanine aminotransferase (aka glutamate pyruvate transaminase, GPT)
Aralar: aspartate–glutamate antiporter SLC25A12 (AGC1)
AST: aspartate aminotransferase (aka glutamate oxaloacetate transaminase, GOT)
ATGL: adipose triglyceride lipase
BCAA: branched-chain amino acid
BCAT2: branched-chain amino acid transferase 2, mitochondrial
BCKA: branched-chain ketoacid
BCKDH: branched-chain ketoacid dehydrogenase
Ca_L_: voltage-dependent Ca^2+^ channels of L-type
CaMKII: Ca^2+^/calmodulin-dependent protein kinase II
CAT: carnitine acyl transferase
Ca_V_: voltage-dependent Ca^2+^ channels
CGI: comparative gene identification 58, ATGL co-activator (aka ABDH5)
Cit C: citrate carrier, mitochondrial
CoA: coenzyme-A
CS: citrate synthase
DAG: diacylglycerol
DAT, DGAT: diacylglycerol *O*-acyltransferase
EPAC: exchange proteins directly activated by cAMP
ER: endoplasmic reticulum
ERK: extracellular regulated kinase
ETF: electron transfer flavoprotein
ETF:QOR: electron transfer flavoprotein:quinone oxidoreductase
F6P: fructose-6-phosphate
FA: fatty acid
FASIS: fatty acid-stimulated insulin secretion
FASN: fatty acid synthase
FH: fumarate hydratase
FUM: fumarate
G6P: glucose-6-phosphate
G6PDH: glucose-6-phosphate dehydrogenase
GAD: glutamate decarboxylase
GC1: glutamate carrier
GDH: glutamate dehydrogenase
GLP-1: glucagon-like peptide 1
GLP1R: glucagon-like peptide 1 receptor
GLS: glutaminase
GLUT: glucose transporter
glycerol3P: glycerol-3-phosphate
GPAT1,2: glycerol-3-phosphate acyltransferase 1,2
GPAT3,4: glycerol-3-phosphate acyltransferase 3,4 (1-acylglycerol-3-phosphate O-acyltransferase)
GPR: G-protein-coupled receptor
GPT: glutamate pyruvate transaminase
GSIS: glucose-stimulated insulin secretion
HMG-CoA: hydroxymethyl-glutaryl-CoA
HMGCoAL: hydroxymethyl-glutaryl-CoA lyase
HSL: hormone-sensitive lipase
IC: isocitrate
ICS: intracristal space
IDH1: isocitrate dehydrogenase 1, cytosolic NADP^+^ dependent
IDH2: isocitrate dehydrogenase 2, mitochondrial NADP^+^ dependent
IDH3: isocitrate dehydrogenase 3, cytosolic NAD^+^ dependent
IF1: ATPase inhibitory factor 1 (*i.e*., ATP synthase inhibitory factor 1)
IGV: insulin granule vesicle
IMM: inner mitochondrial membrane
IP3: inositol-1,4,5-triphosphate
IP3R: inositol-1,4,5-triphosphate receptor
iPLA2γ: Ca^2+^-independent phospholipase A2 isoform γ (PNPLA8)
K_ATP_: ATP-sensitive K^+^ channel
KIC: 2-ketoisocaproate
KIV: 2-ketoisovalerate
KMV: 2-ketoisomethylvalerate
LysoPhA: lysophosphatidic acid
MAG: monoacylglycerol
MAL: malate
MAS: malate/aspartate shuttle
MCU: mitochondrial calcium uniporter
MDH: malate dehydrogenase
ME1: malic enzyme 1, cytosolic
ME3: malic enzyme 3, mitochondrial
MODY: maturity-onset diabetes of the young
NBF: nucleotide-binding fold
NCLX: mitochondrial sodium calcium exchanger
NNT: nicotinamide nucleotide transhydrogenase
NOX4: NADPH oxidase 4
NSCC: nonspecific calcium channels
OAA: oxaloacetate
OCR: oxygen consumption rate
OMM: outer mitochondrial membrane
Orai1: calcium release-activated calcium modulator 1
OXPHOS: oxidative phosphorylation
PC: pyruvate carboxylase
PDH: pyruvate dehydrogenase
PEP: phosphoenolpyruvate
PEPCK2: phosphoenolpyruvate-carboxykinase 2
PhA: phosphatidic acid
PI: pancreatic islets
PIP_2_: phosphatidylinositol 4,5-bisphosphate
PK: pyruvate kinase
PKA: protein kinase A
PKC: protein kinase C
PKL: pyruvate kinase isoform L
PKM2: pyruvate kinase isoform recruitable M2
PLC: phospholipase C
PMCA: plasma membrane Ca^2+^ATPase
PPP: pentose phosphate pathway
PyrC: pyruvate carrier, mitochondrial
Q: ubiquinone
QH_2_: ubiquinol
Rap2: Ras-related protein 2
RC: respiratory chain
RyR: ryanodine receptor
sAC: soluble adenylyl cyclase
S-CoA: succinyl-CoA
SCoA:3oxoAcCoAT: succinyl-CoA:3-ketoacid-CoA transferase
SERCA: sarco/endoplasmic reticulum Ca^2+^-ATPase
SNAP-25: synaptosomal nerve-associated protein 25
SNARE: soluble N-ethylmaleimide-sensitive factor (NSF) attachment protein receptor
SUCC: succinate
SUR1: sulfonylurea receptor 1
TG: triglycerides
tmAC: transmembrane adenylyl cyclase
TRPC: transient receptor potential canonical
TRPM: transient receptor potential melastin
TRPV: transient receptor potential vanilloid
